# The E–Id protein axis modulates the activities of the PI3K–AKT–mTORC1–Hif1a and c-myc/p19Arf pathways to suppress innate variant T_FH_ cell development, thymocyte expansion, and lymphomagenesis

**DOI:** 10.1101/gad.255331.114

**Published:** 2015-02-15

**Authors:** Masaki Miyazaki, Kazuko Miyazaki, Shuwen Chen, Vivek Chandra, Keisuke Wagatsuma, Yasutoshi Agata, Hans-Reimer Rodewald, Rintaro Saito, Aaron N. Chang, Nissi Varki, Hiroshi Kawamoto, Cornelis Murre

**Affiliations:** 1 Department of Molecular Biology, University of California at San Diego, La Jolla, California 92093, USA;; 2 Department of Biochemistry and Molecular Biology, Shiga University of Medical School, Shiga 520-2192, Japan;; 3 Division of Cellular Immunology, German Cancer Research Center, D-69120 Heidelberg, Germany;; 4 Department of Medicine, University of California at San Diego, La Jolla, California 92093, USA;; 5 Center for Computational Biology, Institute for Genomic Medicine, University of California at San Diego, La Jolla, California 92093, USA;; 6 Department of Pathology, University of California at San Diego, La Jolla, California 92093, USA;; 7 Department of Immunology, Institute for Frontier Medical Sciences, Kyoto University, Kyoto 606-8507, Japan

**Keywords:** Id proteins, T-cell quiescence, T-cell lymphoma,, tumor suppressor, FOXO/mTORC1 pathway, c-Myc/p19Arf

## Abstract

It is now well established that the E and Id protein axis regulates multiple steps in lymphocyte development. However, it remains unknown how E and Id proteins mechanistically enforce and maintain the naïve T-cell fate. Here we show that Id2 and Id3 suppressed the development and expansion of innate variant follicular helper T (T_FH_) cells. Innate variant T_FH_ cells required major histocompatibility complex (MHC) class I-like signaling and were associated with germinal center B cells. We found that Id2 and Id3 induced *Foxo1* and *Foxp1* expression to antagonize the activation of a T_FH_ transcription signature. We show that Id2 and Id3 acted upstream of the Hif1a/Foxo/AKT/mTORC1 pathway as well as the c-myc/p19Arf module to control cellular expansion. We found that mice depleted for *Id2* and *Id3* expression developed colitis and αβ T-cell lymphomas. Lymphomas depleted for *Id2* and *Id3* expression displayed elevated levels of *c-myc*, whereas *p19Arf* abundance declined. Transcription signatures of *Id2*- and *Id3*-depleted lymphomas revealed similarities to genetic deficiencies associated with Burkitt lymphoma. We propose that, in response to antigen receptor and/or cytokine signaling, the E–Id protein axis modulates the activities of the PI3K–AKT–mTORC1–Hif1a and c-myc/p19Arf pathways to control cellular expansion and homeostatic proliferation.

## Abstract (precis)

Miyazaki et al. show that Id2 and Id3 suppress the development and expansion of innate variant T_FH_ cells by acting upstream of the Hif1a/Foxo/AKT/mTORC1 pathway as well as the c-myc/p19Arf module. Mice depleted for Id2 and Id3 expression developed colitis and αβ T-cell lymphomas, and the transcription signatures of Id2- and Id3-depleted lymphomas revealed similarities to genetic deficiencies associated with Burkitt lymphoma.

The differentiation of T-lineage cells is initiated in the thymus. Soon after arriving in the thymus, T-cell progenitors initiate TCRβ locus rearrangement, undergo limited expansion, and initiate a T-lineage-specific program of gene expression. This developmental stage is characterized by the lack of expression of the coreceptors CD4 and CD8 and is commonly referred to as the double-negative (DN) stage. Once a functional TCRβ chain has been generated and the pre-TCR is assembled and expressed, DN cells undergo rapid expansion and give rise to CD4^+^CD8^+^ double-positive (DP) cells (Carpenter and Bosselut 2010). Upon reaching the DP compartment, thymocytes exit the cell cycle, initiate TCRα locus rearrangement, and undergo positive and negative selection (Singer et al. 2008; Kreslavsky et al. 2010). The lymphoid populations can be segregated into adaptive or innate immune cells. The selection process permits the developmental progression of a selected group of adaptive T-lineage cells that have acquired a TCR with moderate affinity for major histocompatibility complex (MHC) class II (CD4 single positive [CD4SP]) or class I (CD8SP) associated with self-antigens (Klein et al. 2014). On the other hand, innate T-lineage cells are selected by CD1 for invariant natural killer T (iNKT) cells and by MHC-related protein MR1 for mucosal-associated invariant T lymphocytes (MAIT) (Bendelac et al. 2007; Gold and Lewinsohn 2013)

Adaptive B and T cells express an enormously diverse antigen receptor repertoire. They maintain a naïve lymphoid cell state until they encounter invading pathogens, upon which they expand and differentiate into effector cells. Innate lymphoid cells (ILCs) carry germline-encoded receptors or express a limited antigen receptor repertoire and have the potential to rapidly induce cytokine expression (Cerutti et al. 2013; Verykokakis et al. 2014). The innate lymphoid system is comprised of multiple cell types, including natural killer (NK), lymphoid tissue inducer (LTi), type 2 ILCs, and innate-like B and T cells (Diefenbach et al. 2014). The innate-like B- and T-cell compartment consists of marginal zone B cells, iNKT cells, MAIT cells, and subsets of γδ T cells. ILCs act primarily by modulating the activities of adaptive immune cells.

It is now established that a large majority of developmental trajectories in the thymus involve regulation by members of the helix–loop–helix (HLH) family (Rothenberg 2014). These include E proteins as well as Id proteins. Four E proteins have been identified and characterized. They include E12, E47, HEB, and E2-2. E12 and E47 are encoded by the E2A locus and are generated by differential splicing. E protein DNA-binding activity is regulated by the Id gene products, named Id1–4. Id proteins contain an HLH dimerization domain but lack the basic DNA-binding region. They function predominantly by antagonizing the DNA-binding activities of E proteins (Benezra et al. 1990; Lazorchak et al. 2005; Miyazaki et al. 2014).

E protein levels are abundant in T-cell progenitors, where they activate TCRβ V(D)J locus rearrangement and induce the expression of genes encoding for members of the Notch and pre-TCR signaling cascade (Ikawa et al. 2006; Agata et al. 2007). E47 expression declines in resting DP cells and decreases further upon maturating beyond the TCR checkpoint (Engel et al. 2001; Miyazaki et al. 2011). High E2A expression prevents developmental progression, whereas decreasing E2A and HEB levels promote positive selection (Rivera et al. 2000; Jones and Zhuang 2007). HEB acts in the DP compartment to promote the development of NKT cells (D’Cruz et al. 2010). The traversal of these checkpoints in response to pre-TCR, γδ TCR, and αβ TCR signals is facilitated by the induction of Id3 expression. Beyond the pre-TCR, γδ TCR, and αβ TCR checkpoints, Id3 expression is required to maintain the naïve state (Verykokakis et al. 2010, 2014; Miyazaki et al. 2011; Li et al. 2013). The combined activities of Id2 and Id3 are required to promote efficient developmental progression of CD8SP cells (Jones-Mason et al. 2012).

Here we examined how Id2 and Id3 act mechanistically beyond the TCR checkpoint to orchestrate T-cell fate. We found that the activation of *Id3* and *Id2* expression in DP cells is sequential and that Id2 and Id3 suppressed the development and expansion of innate variant follicular helper T (T_FH_)-like cells acting in turn to promote the ectopic development of germinal center (GC) B cells. The innate T_FH_-like cells carried a highly restricted antigen receptor repertoire indicative of a self-renewing population. We identified a genetic network involving the Id–E protein, AKT–FOXO–mTOR, and Myc–p19Arf modules, which orchestrate a self-renewal-specific program of gene expression. Finally, mice depleted for *Id2* and *Id3* in T cells developed colitis as well as T-cell lymphoma. Collectively, these data point to a regulatory circuitry that underpins the mechanism by which Id2 and Id3 act to antagonize an innate variant T_FH_-specific program of gene expression, maintain thymocyte quiescence, and suppress the development of lymphoma.

## Results

### Expression patterns of Id2 and Id3 in positively selected thymocytes

**Figure 1.**
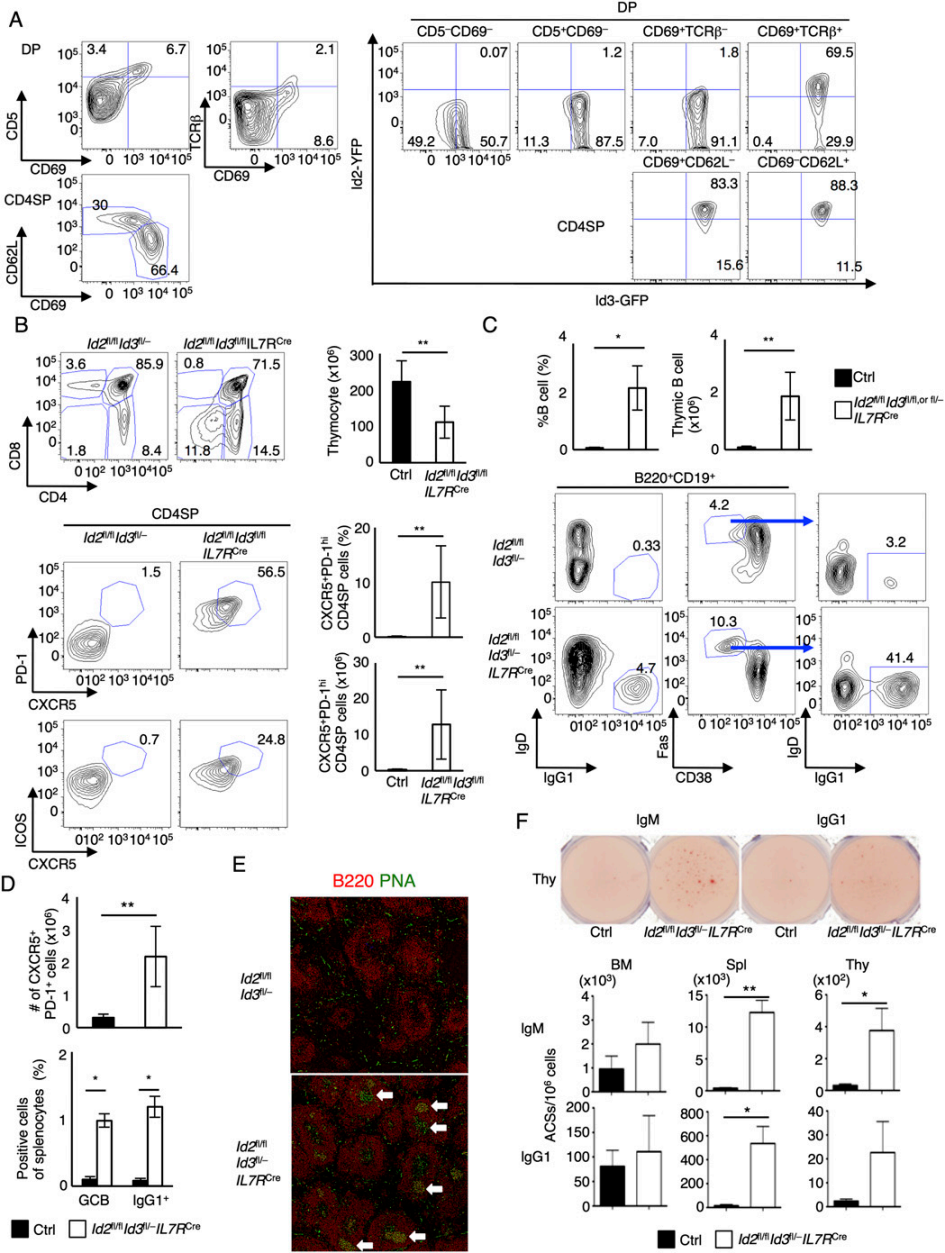
Development of CXCR5^+^PD-1^+^ αβ T cells and IgG1 class-switched B cells in thymi derived from *Id2*^fl/fl^*Id3*^fl/f*l*^*IL7R*^Cre^ mice. (*A*) Flow cytometric analysis of CD69 versus CD5 expression and CD69 versus TCRβ expression gated on CD4^+^CD8^+^ DP cells (*top left*); CD69 versus CD62L expression gated on CD4SP (CD4^+^CD8^−^TCRβ^+^) cells (*bottom left*); and GFP versus YFP expression gated on CD5^−^CD69^−^, CD5^+^CD69^−^, CD69^+^TCRβ^−^, CD69^+^TCRβ^+^ (DP), and CD69^+^CD62L^−^ and CD69^−^CD62L^+^ (CD4SP) cells (*right*). Numbers in quadrants indicate percentages of cells in each compartment. Data are representative of two independent experiments. (*B*, *top left*) Flow cytometric analysis of CD4 versus CD8 expression in total thymocytes derived from 5-wk-old *Id2*^fl/fl^*Id3*^fl/fl^*IL7R*^Cre^ and *Id2*^fl/fl^*Id3*^fl/−^ control mice. (*Top right*) The graph shows the number of total thymocytes of 5-wk-old *Id2*^fl/fl^*Id3*^fl/fl^*IL7R*^Cre^ and *Id2*^fl/fl^*Id3*^fl/−^ control mice. The *bottom* panels show the expression of CXCR5 and PD-1 (*middle*) and CXCR5 and ICOS (*bottom*) gated on CD4SP (CD4^+^CD8^−^TCRβ^+^) cells. The absolute number and frequency of gated cells are shown in adjacent panels. Numbers in plots refer to CXCR5^+^PD-1^+^ and CXCR5^+^ICOS^+^ cells. Data represent the mean ± SD from two independent experiments analyzing four 5-wk-old mice. (*C*) The *top* graphs show the percentage and absolute number of B cells (B220^+^CD19^+^) in thymi derived from 5-wk-old *Id2*^fl/fl^*Id3*^fl/fl^*IL7R*^Cre^ and littermate control mice. Flow cytometric analysis of IgG1 versus IgD expression and CD38 versus Fas expression gated on the B220^+^CD19^+^ population. The *bottom right* panel shows IgG1 and IgD expression gated on the CD38^−^Fas^hi^ cells. Data represent the mean ± SD from two independent experiments analyzing four 5-wk-old mice. (*D*) The absolute number of CXCR5^+^PD-1^+^ CD4T cells and frequency of GC (Fas^+^GL7^+^) B cells and IgG1 class-switched (IgG1^+^IgD^−^) B cells in spleens derived from 5-wk-old *Id2*^fl/fl^*Id3*^fl/fl^*IL7R*^Cre^ mice. Data represent the mean ± SD from two independent experiments analyzing four 5-wk-old mice. (*E*) Representative immunofluorescence staining with anti-B220 antibody and PNA in spleens derived from 5-wk-old *Id2*^fl/fl^*Id3*^fl/fl^*IL7R*^Cre^ mice. Arrows indicate PNA^+^ GCs. (*F*) Representative ELISPOT wells using thymocytes (*top*) and the frequency of IgM- or IgG1-secreting cells in bone marrow (BM), spleen (Spl), and thymus (Thy) derived from 6-mo-old *Id2*^fl/fl^*Id3*^fl/fl^*IL7R*^Cre^ and littermate control mice (*middle* and *bottom*) are shown. (*E*) Data are representative of two independent experiments with three mice each. (*) *P* < 0.05; (**) *P* < 0.01 (Student’s *t*-test).

Previous studies have demonstrated that *Id3* expression is induced at the pre-TCR checkpoint and further elevated during the positive selection process, whereas *Id2* expression is low in positively selected DP cells but elevated in CD4SP or CD8SP cells (Bain et al. 2001; Engel et al. 2001; Miyazaki et al. 2011; Jones-Mason et al. 2012). To examine in greater detail how *Id2* and *Id3* expression is regulated during positive selection, we used *Id2*-YFP and *Id3*-GFP reporter mice (Yang et al. 2011a). Positively selected cells, identified as CD5^+^CD69^−^ or CD69^+^TCRβ^−^ DP cells, expressed higher levels of *Id3* but did not display significant levels of *Id2-*YFP (Fig. 1A). *Id2* expression was only detectable in TCRβ^+^ DP cells (Fig. 1A). The majority of mature CD62L^+^ CD4SP or CD8SP cells displayed abundant levels of *Id2* and *Id3* expression (Fig. 1A). Collectively, these data indicate that the induction of *Id2* and *Id3* expression during positive selection is sequential: *Id3* expression is activated by TCR signaling in positively selected cells, whereas *Id2* expression is induced at a later stage by a separate pathway, which remains to be revealed.

### Development of T_FH_-like cells and GC formation in primary and peripheral lymphoid organs derived from Id2^fl/fl^Id3^fl/fl^IL7R^Cre^ mice

Previous studies have demonstrated key roles of E and Id proteins in enforcing and modulating the pre-TCR and TCR checkpoints (Bain et al. 1999; Engel et al. 2001; D’Cruz et al. 2010; Miyazaki et al. 2011; Jones-Mason et al. 2012). To evaluate the roles of Id2 and Id3 throughout thymocyte development, we used *Id2*^fl/fl^*Id3*^fl/fl^*IL7R*^Cre^ mice to ablate Id2 and Id3 expression in common lymphoid progenitors (CLPs) (Schlenner et al. 2010; Jones-Mason et al. 2012; Niola et al. 2012). Consistent with previous observations, the CD8SP compartment was virtually absent in *Id2*^fl/fl^*Id3*^fl/fl^*IL7R*^Cre^ mice (Fig. 1B; Jones-Mason et al. 2012). Additionally, *Id2*^fl/fl^*Id3*^fl/fl^*IL7R*^Cre^ mice displayed an increase in the total number of DN cells that expressed TCRβ (Supplemental Fig. 1A). Since a previous study showed aberrant development of T_FH_-like cells in thymi derived from *Id3*-null mutant mice, we examined *Id2*^fl/fl^*Id3*^fl/fl^*IL7R*^Cre^ mice for CXCR5, PD-1, and ICOS expression (Miyazaki et al. 2011). We found that a large fraction of CD4SP and DN TCRβ^+^ as well as iNKT cells expressed CXCR5, PD-1, and ICOS in thymi derived from 5-wk-old *Id2*^fl/fl^*Id3*^fl/fl^*IL7R*^Cre^ mice (Fig. 1B; Supplemental Fig. 1A,B). CXCR5^+^PD-1^+^ T_FH_-like cells were detectable in *Id3*^−/−^, *Id3*^fl/−^*CD4*^Cre^, and *Id3*^fl/fl^*IL7R*^Cre^ mice but not in *Id2*^fl/fl^*IL7R*^Cre^ mice (Supplemental Fig. 1C,D). The T_FH_-like populations were accompanied by increased numbers of B220^+^CD19^+^ thymic B cells, Fas^hi^CD38^−^ GC B cells, and IgG1 class-switched cells (Fig. 1C). We found that the segregation of cortical and medullary regions was completely abolished, and B220^+^ cells were observed throughout the thymi derived from 5-wk-old *Id2*^fl/fl^*Id3*^*f*l/fl^*IL7R*^Cre^ mice (Supplemental Fig. 1E). In addition, we noted spontaneous GCs in the spleens derived from *Id2*^fl/fl^*Id3*^fl/fl^*IL7R*^Cre^ mice, accompanied by fewer naïve CD4 T cells and an increased number of T_FH_ cells (Fig. 1D,E; Supplemental Fig. 2A,B). The elevated number of T_FH_ cells was associated with the development of GC B cells, IgG1 class-switched B cells, plasma blasts, and plasma cells (Fig. 1D; Supplemental Fig. 2C). Furthermore, substantial numbers of IgM- and IgG1-secreting cells were detected in thymi and spleens, but not in the bone marrow, derived from 4- to 6-mo-old *Id2*^fl/fl^*Id3*^*f*l/fl^*IL7R*^Cre^ mice (Fig. 1F). Taken together, these data indicate that *Id2* and *Id3* suppress the development and/or selection of T_FH_-like cells and GC B cells in primary and peripheral lymphoid organs.

### Development of innate T_FH_-like cells in Id2^fl/fl^Id3^fl/fl^IL7R^Cre^ mice

**Figure 2.**
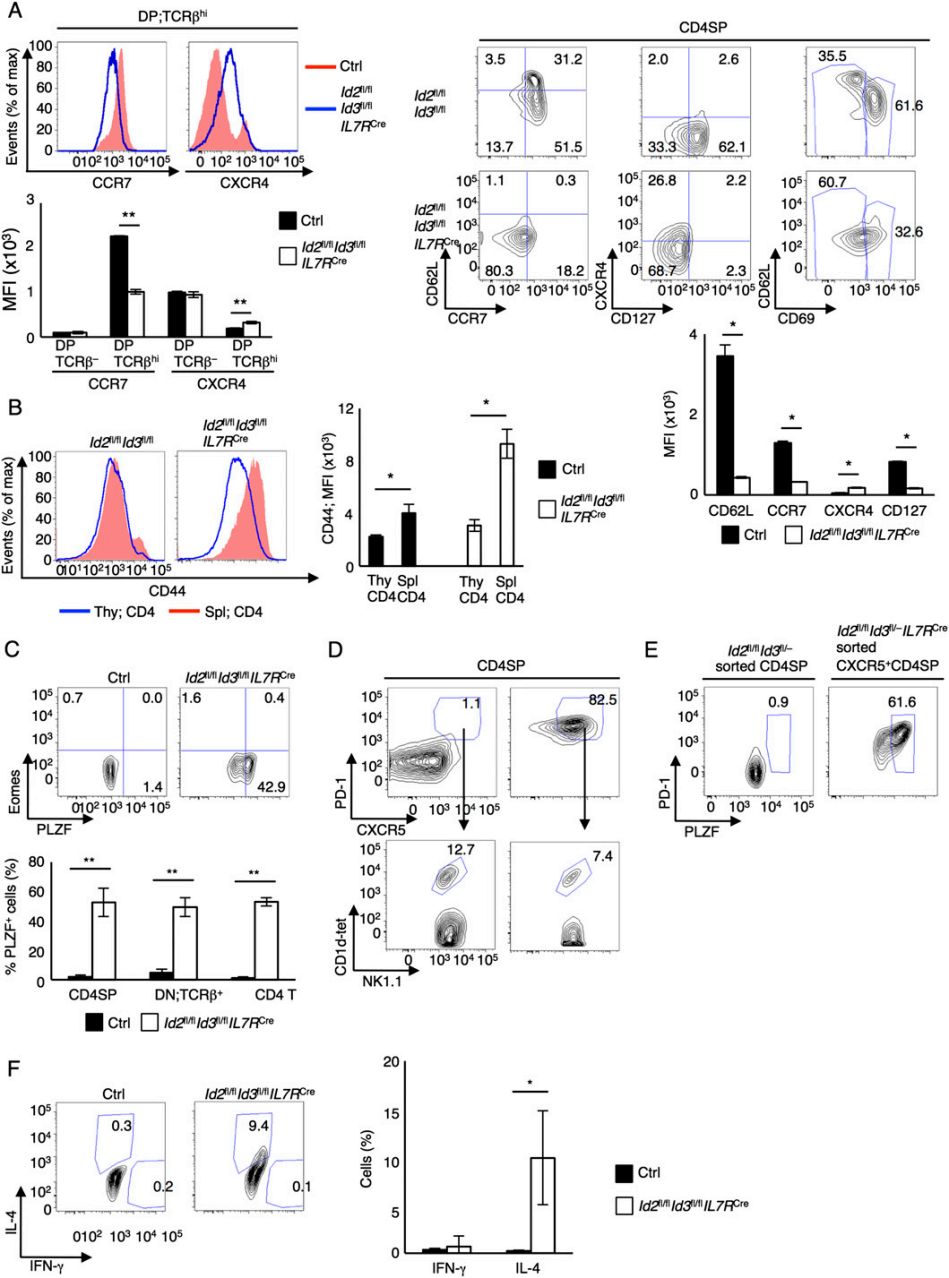
Id2 and Id3 suppress the development of PLZF-expressing non-iNKT αβ T cells. (*A*, *left*) Flow cytometric analysis of CCR7 and CXCR4 gated on TCRβ^+^ DP cells derived from 6-wk-old *Id2*^fl/fl^*Id3*^fl/fl^*IL7R*^Cre^ and littermate control mice. (*Bottom left*) The graph shows CCR7 and CXCR4 expression in CD69^−^TCRβ^−^ and TCRβ^+^ DP cells presented as mean fluorescence intensity (MFI). Flow cytometric analysis of CCR7 versus CD62L, CD127 versus CXCR4, and CD69 versus CD62L gated on CD4SP cells derived from 6-wk-old *Id2*^fl/fl^*Id3*^fl/fl^*IL7R*^Cre^ and littermate control mice. Numbers adjacent to the outlined areas or in quadrants indicate the percentage of cells in the population that was examined. (*Bottom right*) The graph shows the CD62L, CCR7, CXCR4, and CD127 expression in CD4SP cells, presented as MFI. Data are representative of one experiment with 6-wk-old mice (mean ± SD; *n* = 3). (*B*) Flow cytometric analysis of CD44 expression in CD4SP thymocytes (blue) and splenic CD4 T cells (red) derived from 6- to 8-wk-old *Id2*^fl/fl^*Id3*^fl/fl^*IL7R*^Cre^ and *Id2*^fl/fl^*Id3*^fl/fl^ control mice. The *right* panel shows the expression of CD44 in CD4SP thymocytes and splenic CD4 T cells, presented as MFI. Data are representative of one experiment with 6-wk-old mice (mean ± SD; *n* = 4). (*C*, *top*) Flow cytometric analysis of PLZF versus Eomes expression gated on CD4SP cells derived from *Id2*^fl/fl^*Id3*^fl/fl^*IL7R*^Cre^ and control thymus. (*Bottom*) The graph shows the percentage of PLZF-expressing cells in CD4SP, DN;TCRβ^+^ in the thymus and CD4 T cells in the spleen. Numbers in quadrants indicate the percentages of cells. Data are representative of three independent experiments with 6-wk-old mice (mean ± SD; *n* = 4 biological replicates). (*D*) Representative flow cytometric analysis of CXCR5 versus PD-1 expression gated on CD4SP cells and CD1d-tet versus TCRβ expression gated on CXCR5^+^PD-1^+^ CD4SP cells derived from 8-wk-old thymus. (*E*) Flow cytometric analysis of PLZF and PD-1 expression in sorted CXCR5^+^ CD4SP cells (CD4^+^CD8^−^TCRβ^+^CD1d-tet^−^CXCR5^+^) from *Id2*^fl/fl^*Id3*^fl/−^*IL7R*^Cre^ thymus and sorted control CD4SP cells. Numbers indicate PLZF^+^ cells. (*F*, *top*) Cytokine expression in CD4SP cells derived from *Id2*^fl/fl^*Id3*^fl/fl^*IL7R*^Cre^ and control thymi. (*Bottom*) The graph shows the percentage of IFN-γ^+^ and IL-4^+^ cells in CD4SP cells. Data are representative of three independent experiments with 6-wk-old mice (mean ± SD; *n* = 3 biological replicates). (*) *P* < 0.05; (**) *P* < 0.01 (Student’s *t*-test).

To examine in greater detail the phenotypes associated with the development of T_FH_-like cells, CD4SP cells were analyzed for the expression of markers associated with maturation and migration. In line with previous studies, we found that TCRβ^hi^ DP and CD4SP thymocytes displayed aberrant CCR7, CXCR4, CD62L, and CD69 expression in *Id2*^fl/fl^*Id3*^*f*l/fl^*IL7R*^Cre^ mice (Fig. 2A; Jones-Mason et al. 2012). The level of CD44 expression displayed by *Id2*^fl/fl^*Id3*^*f*l/fl^*IL7R*^Cre^ CD4SP thymocytes was distinct from that observed for splenic CD4^+^ T cells, suggesting that the CD4SP cells in the thymus were not derived from recirculating CD4^+^ T cells (Fig. 2B). Previous studies have demonstrated that a fraction of CD4SP cells derived from *Id3*^−/−^ thymi aberrantly expressed PLZF or Eomes (Verykokakis et al. 2010, 2013; Miyazaki et al. 2011; Li et al. 2013; D’Cruz et al. 2014). The fraction of thymocytes expressing PLZF was increased in the thymi and spleens derived from *Id2*^fl/fl^*Id3*^*f*l/fl^*IL7R*^Cre^ mice, while Eomes expressors were mostly lacking (Fig. 2C). Since PLZF is the transcription factor that is expressed in innate T cells such as iNKT cells, we examined whether the innate T_FH_-like population was distinct from that of iNKT cells. We found that only a small fraction of CXCR5^+^ CD4SP cells represented iNKT cells and that the majority of CXCR5^+^ CD4SP cells expressed PLZF (Fig. 2D,E). Furthermore, we found significantly increased numbers of IL-4- but not IFN-γ-producing CD4SP cells (Fig. 2F). Taken together, these data indicate that depletion of *Id2* and *Id3* expression at an early developmental stage results in the development of an innate T_FH_-like population in the thymus.

### Rapidly expanding innate T_FH_-like cells in Id2^fl/fl^Id3^fl/fl^IL7R^Cre^ mice

**Figure 3.**
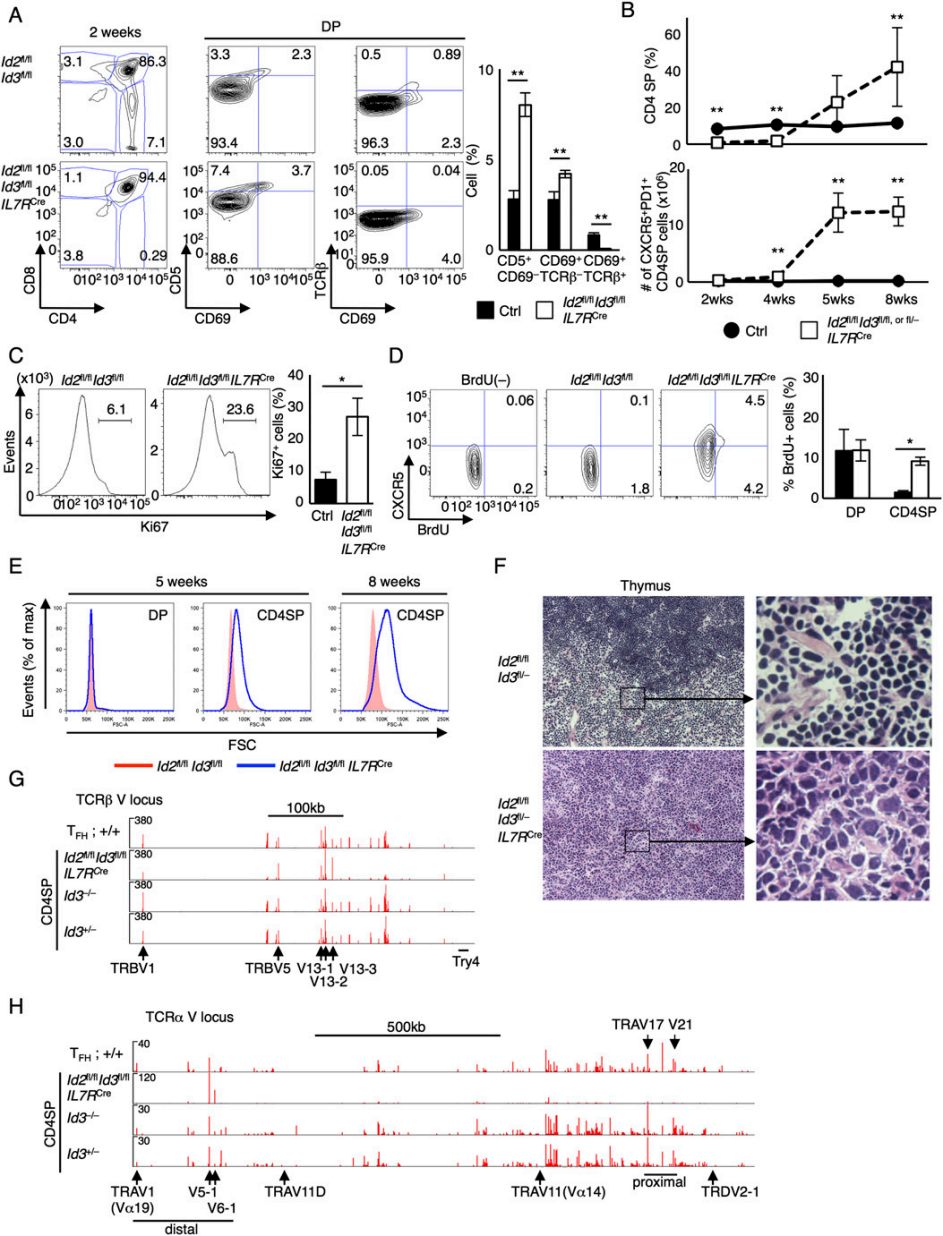
An expanding population of CXCR5^+^ cells in thymi derived from *Id2*^fl/fl^*Id3*^fl/fl^*IL7R*^Cre^ mice. (*A*) Flow cytometric analysis of CD4 versus CD8 expression in total thymocytes and CD69 versus CD5 expression and CD69 versus TCRβ expression gated on DP cells derived from 2-wk-old *Id2*^fl/fl^*Id3*^fl/fl^*IL7R*^Cre^ or littermate control mice. The graph shows the frequency of CD5^+^CD69^−^, CD69^+^TCRβ^−^, and CD69^+^TCRβ^+^ cells in the DP compartment. Numbers adjacent to the outlined areas or in quadrants indicate the percentages of cells in the population. Data are representative of one experiment with 2-wk-old mice (mean ± SD; *n* = 5 [Ctrl] and 3 [*Id2*^fl/fl^*Id3*^fl/fl^*IL7R*^Cre^] biological replicates). (*B*) The frequency of CD4SP cells (*top*) and the number of CXCR5^+^PD-1^+^ CD4SP cells (*bottom*) are plotted. Data were derived from five independent experiments (mean ± SD; *n* = 5 or 3 [2-wk], 3 [4-wk], and 4 [5- or 8-wk] biological replicates). (*C*) Intracellular staining of Ki67 in CD4SP cells derived from 8-wk-old *Id2*^fl/fl^*Id3*^fl/fl^*IL7R*^Cre^ or littermate control mice. Numbers *above* lines indicate percentages of Ki67-expressing cells. The graph shows the frequency of Ki67-expressing cells derived from 6- to 8-wk-old *Id2*^fl/fl^*Id3*^fl/fl^*IL7R*^Cre^ or littermate control mice. Data were derived from two independent experiments (mean ± SD; *n* = 4 biological replicates). (*D*) Flow cytometric analysis of BrdU incorporation and CXCR5 expression gated on CD4SP (TCRβ^+^CD25^−^) cells derived from 8-wk-old *Id2*^fl/fl^*Id3*^fl/fl^*IL7R*^Cre^ or littermate control mice. Mice were injected intraperitoneally with 1 mg of PBS (BrdU^−^) or BrdU twice (24 h and 2 h prior to harvesting). Numbers in quadrants indicate BrdU-incorporated cells. The graph shows the frequency of BrdU^+^ cells in DP and CD4SP cells. Data were derived from two independent experiments (mean ± SD; *n* = 4 biological replicates). (*E*) Flow cytometric analysis of cell size (FSC) in DP and CD4SP cells derived from 5- or 8-wk-old *Id2*^fl/fl^*Id3*^fl/fl^*IL7R*^Cre^ or littermate control mice. Data were derived from two (5-wk) or four (8-wk) independent experiments. (*F*) Representative hematoxylin and eosin (H&E) staining of thymi derived from 6-mo-old *Id2*^fl/fl^*Id3*^fl/fl^*IL7R*^Cre^ or littermate control mice. Original magnification, 200×. Data are representative of three independent experiments. (*G*,*H*) RNA-seq data for the TCRα V locus (*H*) and TCRβ V locus (*G*). mRNA was isolated from sorted CXCR5^+^CD44^hi^ CD4 T cells from SRBC-immunized wild-type spleens (T_FH_) and CD4SP thymocytes derived from 8-wk-old *Id2*^fl/fl^*Id3*^fl/fl^*IL7R*^Cre^, *Id3*^−/−^, and *Id3*^+/−^ thymi and analyzed using RNA-seq. Numbers of reads are indicated for each of the tracks. (*G*,*H*) Two independent experiments with three mice each. (*) *P* < 0.05; (**) *P* < 0.01 (Student’s *t*-test).

To obtain further insight into the mechanism that underpins the roles of Id2 and Id3 in thymocyte development, *Id2*^fl/fl^*Id3*^*f*l/fl^*IL7R*^Cre^ thymi were examined for abnormalities upon aging. We found that at 2 wk after birth, thymi derived from *Id2*^fl/fl^*Id3*^*f*l/fl^*IL7R*^Cre^ mice displayed a complete block in positive selection, with a pronounced defect during the CD69^+^TCRβ^−^-to-CD69^+^TCRβ^hi^ transition (Fig. 3A). For comparison, we analyzed DP cells derived from *TCRα*^−/−^ mice for CD5 or CD69 expression. We found that DP cells derived from *TCRα*^−/−^ thymi did not display either CD69 or CD5 expression (Supplemental Fig. 3A). These data suggest that DP thymocytes that have received a TCR signal required Id2 and Id3 expression to fully differentiate into the TCRβ^hi^ stage. However, unlike the complete block in positive selection observed in 2-wk-old mice, we found that in adult (4- to 8-wk-old) mice, CD4SP cells in *Id2*^fl/fl^*Id3*^*f*l/fl^*IL7R*^Cre^ mice readily were detectable and continued to expand (Fig. 3B, top; Supplemental Fig. 3B). A significant fraction of the expanding CD4SP populations displayed CXCR5 and PD-1 expression (Fig. 3B, bottom; Supplemental Fig. 3B). The expanding CD4SP population showed relatively high levels of Ki67 expression and BrdU incorporation, whereas proliferating cells in the control CD4SP compartment were barely detectable (Fig. 3C,D). Likewise, DN TCRβ^+^ cells derived from *Id2*^fl/fl^*Id3*^*f*l/fl^*IL7R*^Cre^ mice displayed a substantial increase in the fraction of BrdU^+^ cells (Supplemental Fig. 3C). Annexin V expression was not affected in these populations (Supplemental Fig. 3D). We also observed a substantial increase in cell size across the CD4SP but not in the DP compartment in *Id2*^fl/fl^*Id3*^*f*l/fl^*IL7R*^Cre^ thymocytes (Fig. 3E). Over time, the majority of thymocytes expressed CXCR5 and PD-1 associated with large cell size (Supplemental Fig. 3E). Sections of thymi derived from 6-mo-old *Id2*^fl/fl^*Id3*^*f*l/fl^*IL7R*^Cre^ mice showed effacement of the cortico–medullary junction due to proliferation of neoplastic cells with large, hyperchromatic, irregular nuclei and scant cytoplasm (Fig. 3F).

To determine whether the expanding population was derived from a small fraction of selected thymocytes, the TCR repertoires were examined using RNA sequencing (RNA-seq). To this end, RNA was isolated from CD4SP cells from *Id3*^+/−^, *Id3*^−/−^, and *Id2*^fl/fl^*Id3*^*f*l/fl^*IL7R*^Cre^ thymi as well as wild-type T_FH_ cells. We found that Vβ and Vα transcripts associated with wild-type T_FH_ cells and CD4SP thymocytes isolated from *Id3*^+/−^ and *Id3*^−/−^ thymi displayed a similar polyclonal Vβ or Vα repertoire (Fig. 3G,H). On the other hand, CD4SP cells derived from *Id2*^fl/fl^*Id3*^*f*l/fl^*IL7R*^Cre^ mice were enriched for TRBV5, TRBV13-2, and TRBV13-3 as well as distally located TRAV5-1 and TRAV6-1 transcripts (Fig. 3G,H). We also observed a substantial enrichment for transcripts encoding 3′ Jα elements (Supplemental Fig. 4A). Vα and Vβ gene usage observed in *Id2*^fl/fl^*Id3*^*f*l/fl^*IL7R*^Cre^ CD4SP cells, however, was not invariant, as described for iNKT or MAIT cells, but rather differed between the various *Id2*^fl/fl^*Id3*^*f*l/fl^*IL7R*^Cre^ mice that were examined (Supplemental Fig. 4B–D; Le Bourhis et al. 2011). To exclude the possibility that the oligclonal TCRα and TCRβ repertoires were caused by aberrant TCR recombination at the DP stage, we examined the CD69^−^TCRβ^−^ DP cells from *Id2*^fl/fl^*Id3*^*f*l/fl^*IL7R*^Cre^ mice for TCRβ and TCRα rearrangements. No obvious differences were detected for TCRβ and TCRα loci in *Id2*^fl/fl^*Id3*^*f*l/fl^*IL7R*^Cre^ DP cells as compared with control cells (Supplemental Fig. 5A,B). Collectively, these observations indicate that Id2 and Id3 suppress the oligoclonal expansion of innate variant T_FH_-like populations.

### Id2 and Id3 suppress the expansion of innate variant T_FH_-like cells beyond the TCR checkpoint

**Figure 4.**
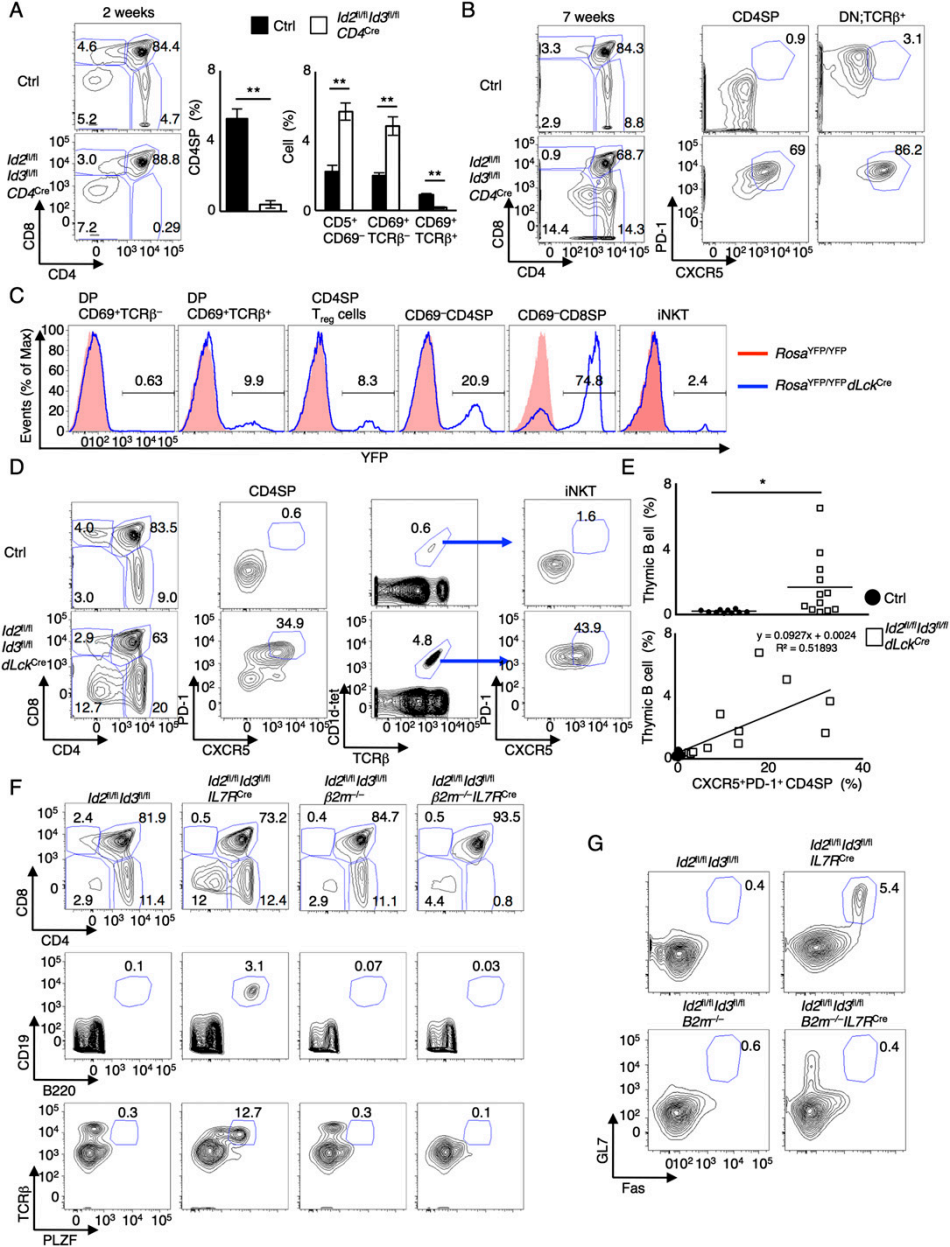
Id2 and Id3 suppress the expansion of innate αβ T cells beyond the TCR checkpoint. (*A*) Flow cytometric analysis of CD4 versus CD8 expression derived from 2-wk-old *Id2*^fl/fl^*Id3*^fl/fl^*CD4*^Cre^ or littermate control mice. Numbers indicate the percentage of cells in each population. Graphs show the frequency of CD4SP cells in total thymocytes and CD5^+^CD69^−^, CD69^+^TCRβ,^−^ and CD69^+^TCRβ^+^ cells gated on the DP compartment. Data are representative of one experiment with 2-wk-old mice (mean ± SD; *n* = 5 biological replicates). (*B*) Flow cytometric analysis of CD4 versus CD8 expression in total thymocytes and CXCR5 versus PD-1 expression gated on CD4SP cells (CD4^+^CD8^−^TCRβ^+^CD1d-tet^−^) or DN;TCRβ^+^ cells (CD4^−^CD8^−^TCRβ^+^CD1d-tet^−^) derived from 7-wk-old *Id2*^fl/fl^*Id3*^fl/fl^*CD4*^Cre^ or littermate control mice. Data were obtained from two independent experiments. (*C*) Representative flow cytometric analysis of YFP expression gated on each compartment derived from *Rosa*^YFP/YFP^ (red) or *Rosa*^YFP/YFP^*dLck*^Cre^ (blue) mice. Numbers *above* the lines indicate percentages of YFP-expressing cells. Data were derived from two independent experiments. (*D*) Flow cytometric analysis of CD4 versus CD8 expression (*left*) and TCRβ versus CD1d-tet expression (*middle right*) in total thymocytes and CXCR5 versus PD-1 expression gated on CD4SP (CD4^+^CD8^−^TCRβ^+^CD1d-tet^−^) cells (*middle left*) or iNKT (CD1d-tet^+^TCRβ^+^) cells (*right*) derived from 10-wk-old *Id2*^fl/fl^*Id3*^fl/fl^*dLck*^Cre^ or littermate control mice. Data were derived from four independent experiments. (*E*) Graphs show the frequencies of thymic B (B220^+^CD19^+^) cells and the correlation between thymic B cells and CXCR5^+^PD-1^+^ CD4SP cells. Data were derived from seven independent experiments. Ten-week-old to 16-wk-old mice were analyzed. (mean ± SD; *n* = 10 [Ctrl] and 12 [*Id2*^fl/fl^*Id3*^fl/fl^*dLck*^Cre^] biological replicates). (*F*) Flow cytometric analysis of CD4 versus CD8 expression, B220 versus CD19 expression, and PLZF versus TCRβ expression in total thymocytes derived from *Id2*^fl/fl^*Id3*^fl/fl^, *Id2*^fl/fl^*Id3*^fl/fl^*IL7R*^Cre^, *Id2*^fl/fl^*Id3*^fl/fl^*β2m*^*−/−*^, and *Id2*^fl/fl^*Id3*^fl/fl^*β2m*^*−/−*^*IL7R*^Cre^ mice. (*G*) Fas and GL7 expression gated on B220^+^CD19^+^ splenic B cells derived from *Id2*^fl/fl^*Id3*^fl/fl^, *Id2*^fl/fl^*Id3*^fl/fl^*IL7R*^Cre^, *Id2*^fl/fl^*Id3*^fl/fl^*β2m*^*−/−*^, and *Id2*^fl/fl^*Id3*^fl/fl^*β2m*^*−/−*^*IL7R*^Cre^ mice. Numbers adjacent to the outlined areas indicate the percentage of cells in each population. Data are representative of three independent experiments. (*) *P* < 0.05; (**) *P* < 0.01 (Student’s *t*-test).

To deplete *Id2* and *Id3* expression in T-lineage cells, *Id2*^fl/fl^*Id3*^*f*l/*fl*^*CD4-Cre* mice were generated. Similar to as described above for *Id2*^fl/fl^*Id3*^*f*l/fl^*IL7R*^Cre^ mice, at 2 wk after birth, positive selection was severely impaired in *Id2*^fl/fl^*Id3*^*f*l/fl^*CD4*^Cre^ thymi (Fig. 4A). However, 7-wk-old *Id2*^fl/fl^*Id3*^*f*l/fl^*CD4*^Cre^ mice displayed CD4SP, DN TCRβ^hi^, and iNKT populations that expressed high levels of CXCR5, PD-1, ICOS, and PLZF expression (Fig. 4B; Supplemental Fig. 6A,B). Again, these populations were associated with elevated numbers of thymic B cells as well as IgG1 class-switched GC B cells (Supplemental Fig. 6C).

Since recent studies have demonstrated a critical role for *Id2* and *Id3* in regulatory T (T_reg_) cells, it remained possible that the innate T_FH_-like population developed because of systemic inflammatory conditions (Miyazaki et al. 2014). To exclude this possibility and investigate the role of *Id2* and *Id3* in thymocyte development beyond the TCR checkpoint, *Id2*^fl/fl^*Id3*^fl/fl^*dLck*^Cre^ mice were generated (Zhang et al. 2005). We found that Cre-mediated deletion in *dLck*^Cre^ mice was initiated beyond the CD69^+^TCRβ^+^ DP stage (Fig. 4C; Supplemental Fig. 7A). We found that a substantial fraction of peripheral T_reg_ cells did not exhibit Cre activity, indicative of T_reg_ cells expressing wild-type levels of Id2 and Id3 in *dLck*^Cre^ mice (Supplemental Fig. 7A). As observed for *Id2*^fl/fl^*Id3*^*f*l/fl^*Il7R*^Cre^ and *Id2*^fl/fl^*Id3*^*f*l/fl^*CD4*^Cre^ mice, in thymi derived from *Id2*^fl/fl^*Id3*^fl/fl^*dLck*^Cre^ mice, we found increased percentages of CD4SP cells and iNKT cells that expressed CXCR5, PD-1, and PLZF and a variant but limited TCRVα and TCRVβ repertoire (Fig. 4D; Supplemental Fig. 7B–D). Again, we noted an increase in the percentages of thymic B cells and a significant relationship between the proportions of thymic B cells and CXCR5^+^PD-1^+^ CD4SP cells (R^2^ = 0.51893) (Fig. 4E). Notably, we detected transcripts initiated from the first exon of the *Id2* and *Id3* genes in sorted CXCR5^−^PD-1^−^ CD4SP cells but not in sorted CXCR5^+^PD-1^+^ CD4SP cells from identical *Id2*^fl/fl^*Id3*^fl/fl^*dLck*^Cre^ mice, consistent with the notion that the aberrant activation of CXCR5 and PD-1 expression was caused by depletion of *Id2* and *Id3* expression (Supplemental Figure 7E). Taken together, these data indicate that Id2 and Id3 expression is required to suppress the development and expansion of an innate variant T_FH_-like population beyond the TCR checkpoint.

To determine how Id2- and Id3-depleted innate variant T_FH_-like cells are selected, *β2m*^*−/−*^*Id2*^fl/fl^*Id3*^*f*l/fl^*IL7R*^Cre^ mice were generated. We found that the development of the innate variant T_FH_ cell population required β2-microglobulin expression (Fig. 4F). Specifically, CD4SP and CD8SP thymocytes were barely detectable in thymi derived from 8- to 10-wk-old *β2m*^*−/−*^*Id2*^fl/fl^*Id3*^*f*l/fl^*IL7R*^Cre^ mice (Fig. 4F). Likewise, CXCR5^+^PD-1^+^ CD4SP cells, DN TCRβ^+^ cells, PLZF-expressing T cells, and thymic B cells were virtually absent in *β2m*^*−/−*^*Id2*^fl/fl^*Id3*^*f*l/fl^*IL7R*^Cre^ thymi (Fig. 4F; Supplemental Fig. 8A). Depletion of *β2m* expression in *Id2*^fl/fl^*Id3*^*f*l/fl^*Il7R*^Cre^ mice also abolished the development of GC and IgG1 class-switched B cells (Fig. 4G; Supplemental Fig. 8A). Thymi derived from *Id2*^fl/fl^*Id3*^*f*l/fl^*β2m*^*−/−*^*IL7R*^Cre^ mice showed a normal thymic cortex but abnormal medulla (Supplemental Fig. 8B). These data indicate that selection of Id2- and Id3-depleted innate variant T_FH_ cells requires β2-microglobulin expression and that this population is closely associated with the development of GCs even in the absence of immunization.

### Gene expression signature of innate variant T_FH_-like cells

**Figure 5.**
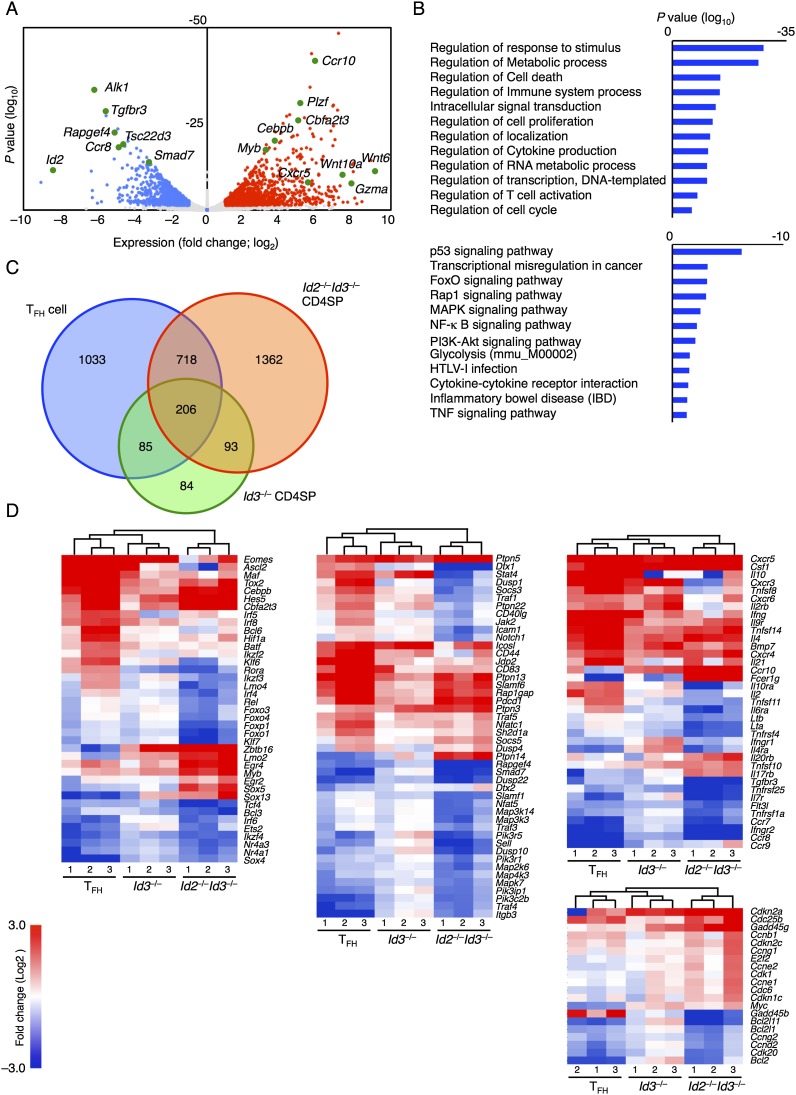
Transcription signature of innate T_FH_-like cells. (*A*) Volcano plot of RNA-seq analysis of mRNA expression in sorted CD4SP thymocytes derived from 8-wk-old littermate control or *Id2*^*fl/fl*^*Id3*^*fl/ll*^*IL7R*^*cre*^ mice. Red dots represent genes up-regulated in *Id2*^fl/fl^*Id3*^fl/fl^*IL7R*^Cre^ CD4SP cells (more than twofold, *P* < 0.05, calculated by raw count value). Blue dots represent genes down-regulated in *Id2*^fl/fl^*Id3*^fl/fl^*IL7R*^Cre^ CD4SP cells (more than twofold, *P* < 0.05). Gray dots represent genes whose expression was not significantly altered. (*B*) Clusters of genes whose expression was modulated in Id2- and Id3-depleted CD4SP thymocytes were identified using GO terms (*top*) and KEGG pathways (*bottom*). *P*-values are shown. (*C*) RNA-seq data of mRNA expression isolated from CXCR5^+^CD44^hi^ CD4T cells from SRBC-immunized wild-type spleen (T_FH_) and CD4SP thymocytes derived from 8-wk-old *Id2*^fl/fl^*Id3*^fl/fl^*IL7R*^Cre^ (*Id2*^−/−^*Id3*^−/−^ CD4SP), *Id3*^*−/−*^ (*Id3*^*−/−*^ CD4SP), and control thymi. Expression was normalized to control CD4SP cells. The Venn diagram shows quantification of genes significantly changed in T_FH_ cells, *Id3*^−/−^ CD4SP cells, and *Id2*^*fl/fl*^*Id3*^*fl/ll*^*IL7R*^*cre*^ CD4SP cells compared with control CD4SP cells. (*D*) Heat maps are presented to visualize differences in gene expression patterns for T_FH_ cells, CD4SP cells sorted from Id3^−/−^ thymocytes, and CD4SP cells sorted from IL7R-Cre;Id2F/F;Id3F/F mice as compared with CD4SP control thymocytes. Bars *above* the heat maps indicate similarities between the different samples presented in each of the lanes. Differentially expressed genes are shown only for those that displayed greater than twofold differences (*P* < 0.05) as compared with control CD4SP thymocytes. The heat maps represent different ontology groups, including genes encoding for factors that regulate transcription (*left*), signal transduction (*middle*), and cell cycle (*bottom right*). (*Top right*) The heat map displays the KEGG pathway for chemokines, cytokines, and associated receptors.

To determine how Id2 and Id3 suppressed the oligoclonal expansion of an innate variant T_FH_-like population, CD4SP cells derived from control and *Id2*^fl/fl^*Id3*^fl/fl^*IL7R*^Cre^ thymi were isolated and analyzed by RNA-seq. Two-thousand-three-hundred-seventy-nine genes were differentially expressed (greater than twofold, *P* < 0.05; 1291 up-regulated; 1088 down-regulated) in *Id*-depleted CD4SP cells (Fig. 5A). Prominent among those genes were *Cxcr5*, *Ccr10*, *Wnt10a*, *Gzma*, *Myb*, *Cebpb*, *Tgfbr3*, and *Smad7* (Fig. 5A). Gene ontology (GO) analysis revealed that a large fraction of differentially expressed transcripts encoded for proteins associated with metabolism, cytokine production, RNA metabolism, T-cell activation, and cell cycle progression (Fig. 5B). Furthermore, Kyoto Encyclopedia of Genes and Genomics (KEGG) pathway analysis revealed p53 and genes associated with the PI3K–AKT, MAPK, and Rap1 pathways as well as genes involved in the suppression of inflammatory bowel disease as being affected by depletion of Id2 and Id3 expression (Fig. 5B). Next, we compared the transcription signatures of Id2/Id3-depleted CD4SP thymocytes with *Id3*^−/−^ CD4SP and peripheral T_FH_ cells. We found that 924 genes showed overlap between T_FH_ and *Id2*- and *Id3*-deficient CD4SP cells as compared with wild-type CD4SP thymocytes. Two-hundred-ninety-nine genes showed overlap between CD4SP cells isolated from *Id3*^−/−^ and *Id2*^fl/fl^*Id3*^fl/fl^*IL7R*^Cre^ thymi, whereas the expression of 1362 genes was modulated only in innate T_FH_-like cells when compared with control CD4SP cells (Fig. 5C; Supplemental Fig. 9A). Transcription signatures derived from *Id3*^−/−^ CD4SP cells showed an intermediate pattern when comparing T_FH_ and CD4SP cells derived from *Id2*^fl/fl^*Id3*^fl/fl^*IL7R*^Cre^ thymi (Fig. 5C). The expression patterns of FcεR1 and IL-17rβ in control and mutant thymocytes were validated using flow cytometry (Supplemental Fig. 9B). Notably, genes associated with T_FH_ cell function were altered across the three populations, including *Maf*, *Batf, Foxo1*, *Foxp1*, *Sh2d1a*, *Cxcr5*, *Il4*, and *Il21* but not *Bcl6* and *Ascl2* (Fig. 5D; Ma et al. 2012; Wang et al. 2014; Xiao et al. 2014). As predicted, the expression levels of genes associated with an innate-specific program of gene expression were also elevated, including *Zbtb16* (*Plzf*), *Myb*, and *Egr2* (Kovalovsky et al. 2008; Hu et al. 2010; Seiler et al. 2012). Finally, consistent with an expanding population of *Id2*- and *Id3*-depleted CD4SP thymocytes, the expression patterns of genes associated with cell cycle regulation were affected (Fig. 5D). Taken together, these observations indicate that gene expression patterns of Id-depleted CD4SP thymocytes overlap, albeit partially, with that of T_FH_ and innate T-lineage cells.

### A genetic circuitry links Id2 and Id3 and the AKT–FOXO–mTORC1 axis

**Figure 6.**
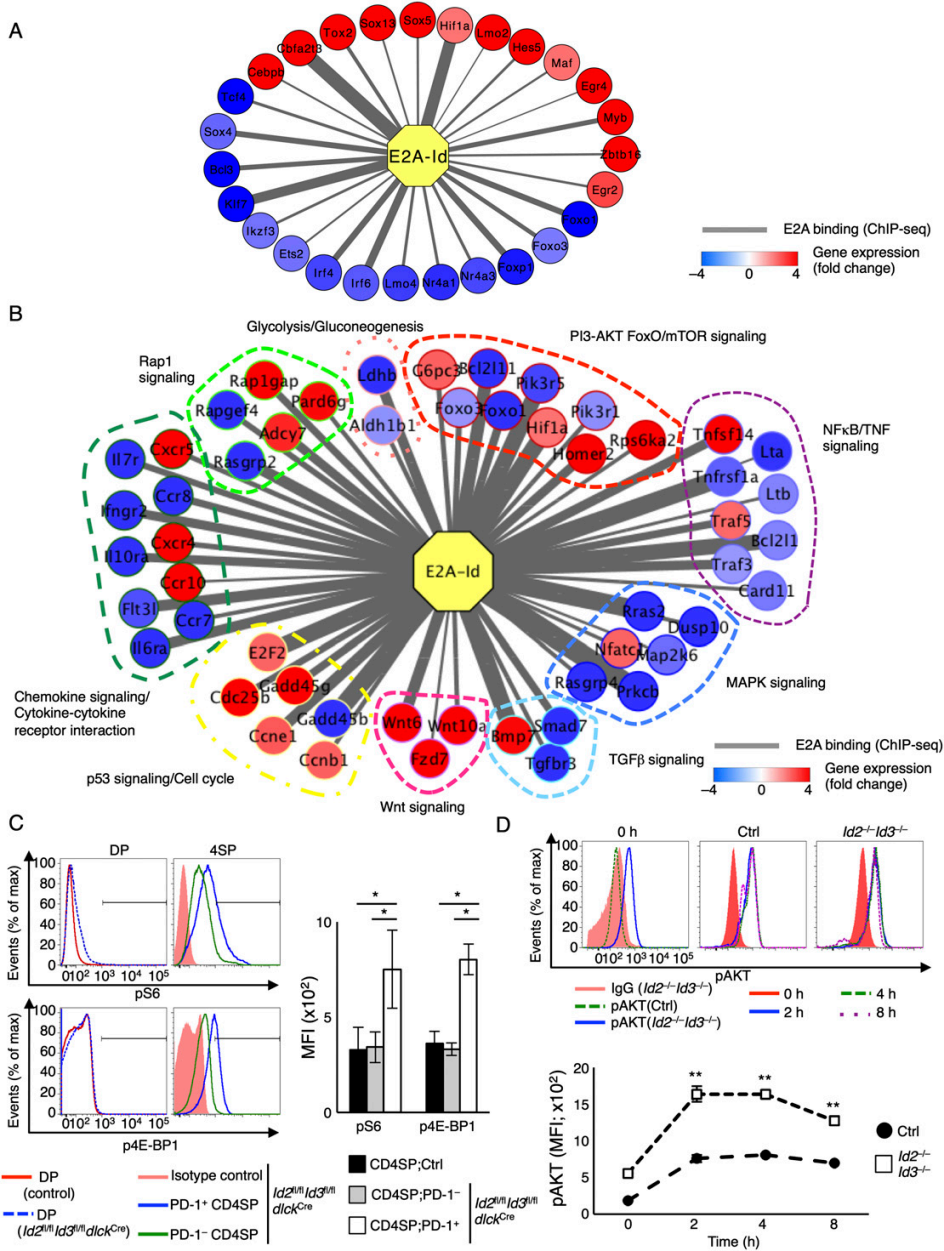
Id2, Id3, and the FOXO/mTOR axis. (*A*) Regulatory network that connects the activity of E proteins and an ensemble of transcriptional regulators in Id2- and Id3-depleted CD4SP thymocytes into a common framework. E2A ChIP-seq (chromatin immunoprecipitation [ChIP] combined with deep sequencing) data were derived from E2A-deficeint T-cell lymphoma cells transduced with E47 (Lin et al. 2010). The widths of the connectors reflect relative peak scores of E2A occupancy. Colors reflect higher (red) or lower (blue) gene expression levels in *Id2*^fl/fl^*Id3*^fl/fl^*IL7R*^Cre^ CD4SP cells as compared with control CD4SP thymocytes. (*B*) Networks that link distinct pathways, as defined by KEGG analysis, to E2A occupancy. (*C*) Flow cytometric analysis of phosphorylation of S6 (pS6) and 4E-BP1 (p4E-BP1) in DP and CD4SP cells for the indicated populations derived from control and *Id2*^fl/fl^*Id3*^fl/fl^*dLck*^Cre^ mice. The graph shows the level of phosphorylated S6 and 4E-BP1, presented as MFI. Data are representative of three independent experiments (mean ± SD; *n* = 3 biological replicates). (*D*) Flow cytometric analysis of phosphorylation of AKT. Sorted CD4SP cells (CD4^+^CD8^−^TCRβ^+^CD1d-tet^−^) derived from the *Id2*^fl/fl^*Id3*^fl/fl^*IL7R*^Cre^ thymus were stimulated with plate-coated anti-CD3ε (2 μg/mL) and anti-CD28 (5 μg/mL) antibodies and analyzed at the indicated time points for pAKT expression. (*) *P* < 0.05; (**) *P* < 0.01 (Student’s *t*-test).

In previous studies, we had identified a spectrum of target genes regulated by the Id*–*E protein module in T-lineage cells, including *Cxcr5*, *Il10*, *Hif1a*, *Ikzf3*, *Myb*, *Il10ra*, *E2f2*, and *Bmp7* (Miyazaki et al. 2014). Here we found additional target genes that are directly regulated by the E*–*Id protein axis, including *Foxo1*, *Foxo3*, *Foxp1*, *Slamf6*, *Il7r*, *Il6ra*, *Cxcr4*, *Tgfbr3*, *Wnt10a*, *Cdc25b*, *Gadd45b*, *Bcl2l1*, *Bcl2l11*, and *Rps6ka2* (Supplemental Fig. 9C; data not shown). To examine for molecular pathways or modules associated with depletion of *Id2* and *Id3* expression, transcription signatures derived from *Id*-depleted CD4SP thymocytes and E protein occupancy were linked into a common framework using Cytoscape software (Fig. 6A). Using this approach, we identified an ensemble of E protein targets—including Foxo1, Foxo3 and Foxp1—associated with the maintenance of a naïve and quiescent state. Additionally, a cluster of target genes was identified closely linked with an innate-like transcription signature, including *Myb*, *Plzf*, *Egr2*, and *Sox13* (Fig. 6A; Melichar et al. 2007; Kerdiles et al. 2009; Feng et al. 2011; Hedrick et al. 2012). Finally, we found that clusters of genes associated with the PI3K–AKT/FoxO/mTOR, NFκB/TNF, MAPK, Rap1, cytokine/chemokine, and p53/cell cycle pathways were targeted by the E and Id protein module (Fig. 6B).

To validate these findings, we examined activation of the mTOR and AKT pathways in developing thymocytes using flow cytometry. We found significant increases in phospho-S6 and phospho-4E-BP1 levels in *Id2*- and *Id3*-depleted PD-1^+^ CD4SP cells derived from *Id2*^fl/fl^*Id3*^fl/fl^*dLck*^Cre^ thymi as compared with control thymi (Fig. 6C). As predicted, phospho-S6 and phospho-4E-BP1 levels were not altered in PD-1^−^ CD4SP or DP cells derived from *Id2*^fl/fl^*Id3*^fl/fl^*dLck*^Cre^ versus control mice (Fig. 6C). To determine whether the E*–*Id protein axis also regulates the activity of the mTORC pathway to control the expansion of innate γδ T cells, Id3^−/−^ Vγ1.1^+^ cells were examined for the expression of phospho-S6 and phospho-4E-BP1. Indeed, Id3^−/−^ Vγ1.1^+^ cells displayed significantly increased levels of phopho-S6 and phospho-4E-BP1 (Supplemental Fig. 10). In contrast, Id3^−/−^ Vγ2^+^ cells were not associated with elevated levels of phopho-S6 and phospho-4E-BP1 (Supplemental Fig. 10). Next, we examined the phosphorylation status of AKT (pAKT) in sorted control and Id-depleted CD4SP thymocytes. We found that resting control CD4SP cells lacked detectable levels of pAKT (Fig. 6D, left panel). However, activating TCR signaling readily elevated levels of pAKT (Fig. 6D, middle and right panels). Notably, *Id2*- and *Id3*-depleted CD4SP cells exhibited pAKT expression in the absence of TCR stimulation, and TCR-mediated signaling resulted in even higher levels of pAKT (Fig. 6D, bottom). Taken together, these data indicate that the E–Id protein axis modulates the PI3K–AKT–FOXO–mTORC1 pathway at multiple steps.

### Id2 and Id3 suppress the development of αβ T-cell lymphomas

**Figure 7.**
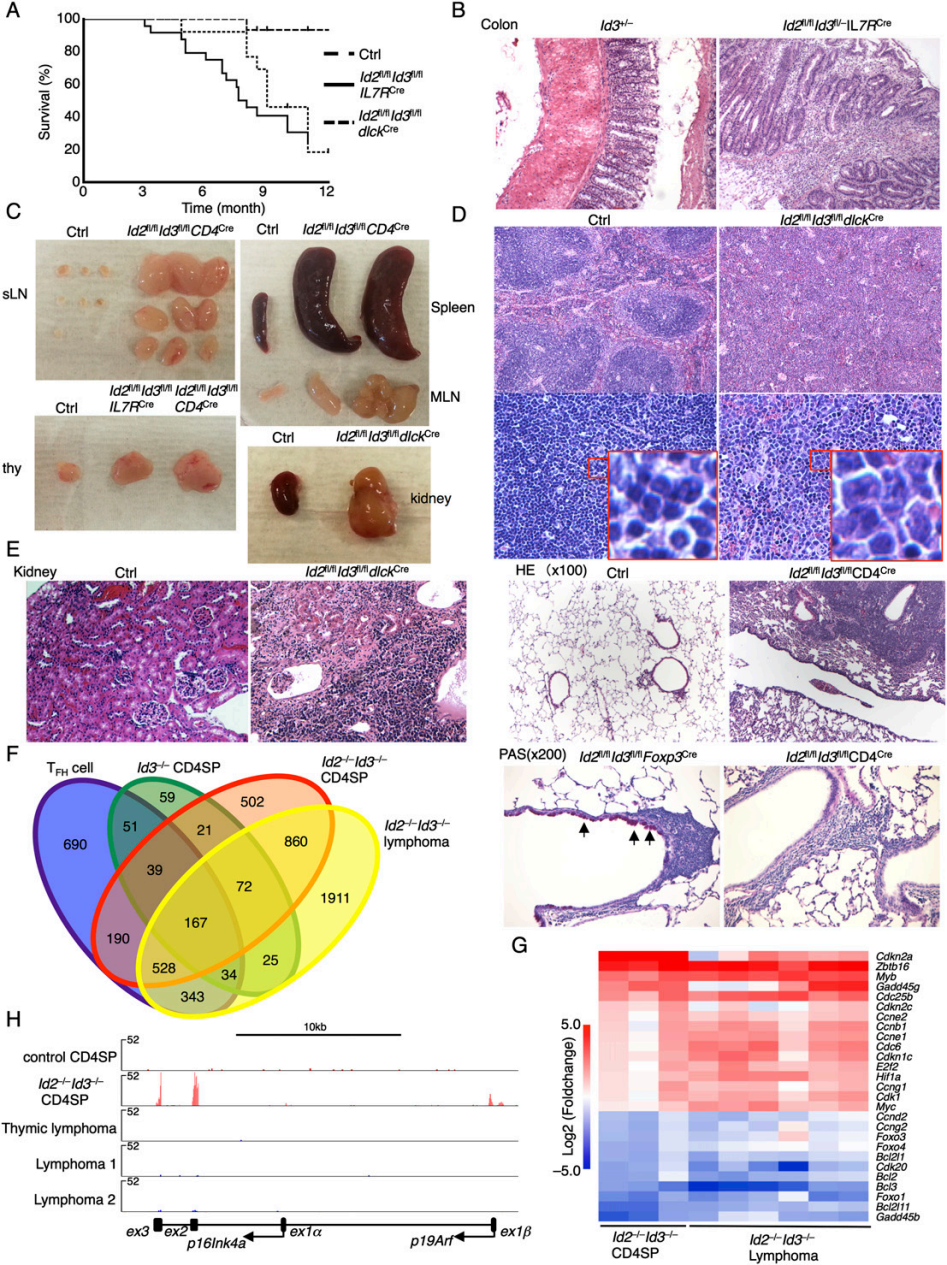
Id2 and Id3 suppress the development of T-cell lymphoma. (*A*) Kaplan-Meier curve of a survival plot of *Id2*^fl/fl^*Id3*^fl/fl^*IL7R*^Cre^, *Id2*^fl/fl^*Id3*^fl/fl^*dLck*^Cre^, and littermate control mice. (*Id2*^fl/fl^*Id3*^fl/fl^*IL7R*^Cre^, *n* = 24; *Id2*^fl/fl^*Id3*^fl/fl^*dLck*^Cre^, *n* = 14; control, *n* = 16). (*B*) Representative H&E staining of colons derived from 6-mo-old *Id3*^+/−^ or *Id2*^fl/fl^*Id3*^fl/−^*IL7R*^Cre^ mice. Original magnification, 100×. (*C*) Representative images of subcutaneous lymph nodes (sLN), thymi, spleens, mesenteric LNs (MLN), and kidneys derived from 8- to 12-mo-old control, *Id2*^fl/fl^*Id3*^fl/fl^*IL7R*^Cre^, *Id2*^fl/fl^*Id3*^fl/fl^*CD4*^Cre^, and *Id2*^fl/fl^*Id3*^fl/fl^*dLck*^Cre^ mice are shown. (*D*) Representative H&E stainings of spleens derived from control and *Id2*^fl/fl^*Id3*^fl/fl^*dLck*^Cre^ mice are displayed. Original magnifications: *top*, 100×; *bottom*, 400×. (*E*) Representative H&E stainings of kidneys (*left*) and lungs (*top right*) derived from control, *Id2*^fl/fl^*Id3*^fl/fl^*dLck*^Cre^, and *Id2*^fl/fl^*Id3*^fl/fl^*CD4*^Cre^ mice are shown. The kidney section was derived from metastasis of lymphoma cells as shown in *C*. (*Bottom right*) PAS stainings of the lungs from *Id2*^fl/fl^*Id3*^fl/fl^*Foxp3*^Cre^ and *Id2*^fl/fl^*Id3*^fl/fl^*CD4*^Cre^ mice are shown. Arrows indicate mucus-producing goblet cells. Original magnifications: kidney, 200×; lung (H&E), 100×; lung (PAS), 200×. (*F*) The Venn diagram shows overlaps among T_FH_ cells, *Id3*^−/−^ CD4SP cells, *Id2*^−/−^*Id3*^−/−^ CD4SP cells, and *Id2*^−/−^*Id3*^−/−^ lymphoma cells as compared with control CD4SP cells, as shown in Figure 5C. (*G*) Heat map for selected significantly differentially expressed genes that are differentially expressed in *Id2-* and *Id3-*depleted CD4SP thymocytes as well as lymphomas. Clusters of genes associated with cell cycle and tumor suppression are indicated. (*H*) Visualized RNA-seq data across the *Cdkn2a* locus, presented in reads per million reads aligned (RPM). Arrows indicate transcription start site of *p19Arf* and *p16Ink4a* gene and direction of transcription.

Previous studies have demonstrated that the development of Burkitt lymphoma is closely associated with mutations across the Id3 HLH region, that forced expression of Id2 in a murine model of BCR–ABL interferes with the development of chronic myeloid leukemia, and that Id3-deficient mice develop γδ T-cell lymphomas (Ko et al. 2008; Li et al. 2010; Schmitz et al. 2012). To determine whether the combined loss of *Id2* and *Id3* expression in T-lineage cells leads to the development of lymphoma, aged *Id2*^fl/fl^*Id3*^fl/fl^*IL7R*^Cre^ mice were monitored for signs of distress. We found that a substantial fraction of 6- to 8-mo-old *Id2*^fl/fl^*Id3*^fl/fl^*IL7R*^Cre^ mice exhibited ruffled fur, hunched posture, and rectal prolapse (data not shown). The majority of *Id2*^fl/fl^*Id3*^fl/fl^*IL7R*^Cre^ or *Id2*^fl/fl^*Id3*^fl/fl^*dLck*^Cre^ mice died within 1 yr (Fig. 7A). Histological analysis showed moderate to severe colitis in 6- to 8-mo-old *Id2*^fl/fl^*Id3*^fl/fl^*IL7R*^Cre^, *Id2*^fl/fl^*Id3*^fl/fl^*CD4*^Cre^, and *Id2*^fl/fl^*Id3*^fl/fl^*dLck*^Cre^ mice (Fig. 7B; Supplemental Fig. 11A). In addition to colitis, we noted splenomegaly and large subcutaneous and/or mesenteric lymph nodes in 21 out of 41 8- to 14-mo-old *Id2*^fl/fl^*Id3*^fl/fl^*IL7R*^Cre^, *Id2*^fl/fl^*Id3*^fl/fl^*CD4*^Cre^, and *Id2*^fl/fl^*Id3*^fl/fl^*dLck*^Cre^ mice (Fig. 7C). Four out of 21 *Id2*^fl/fl^*Id3*^fl/fl^*IL7R*^Cre^, *Id2*^fl/fl^*Id3*^fl/fl^*CD4*^Cre^, and *Id2*^fl/fl^*Id3*^fl/fl^*dLck*^Cre^ mice also developed thymic lymphoma (Fig. 7C; Supplemental Fig. 11B). Histopathological analysis of the peripheral lymphoid organs displayed a disorganized architecture, such as blurring of the demarcation between red and white pulp in the spleen, with infiltrating monomorphic lymphoid cells containing large polymorphic nuclei and scant cytoplasm (Fig. 7D; Supplemental Fig. 11C). The infiltrate in the lungs derived from *Id2*^fl/fl^*Id3*^fl/fl^*CD4*^Cre^ mice was composed of neoplastic nucleated cells (Fig. 7E). We note that lymphocyte infiltration was not caused by inflammatory disease, since there was no evidence of elevated mucin levels in the bronchial epithelium (lack of periodic acid Schiff [PAS]-positive goblet cells) (Fig. 7E). The lymphomas that developed in lymph nodes and spleens were mainly composed of TCRβ^+^CD3^+^ T-lineage cells associated with a CD8^low^CD4^−^, CD8^−^CD4^−^ or CD8^−^CD4^+^ phenotype (Supplemental Fig. 11E). As expected, a large fraction of these cells showed CXCR5 and PD-1 expression (Supplemental Fig. 11E). Finally, we found that lymphoma cells were highly malignant, as evidenced by metastasis across the liver (17 out of 21), kidney (three out of 10), and lung (10 out of 21) tissues (Fig. 7C,E; Supplemental Fig. 11B,D). Collectively, these observations indicate that depletion of Id2 and Id3 in T-lineage cells leads to increased levels of morbidity caused by the development of lymphoma and/or inflammatory disease.

### The c-Myc and p19Arf axis and the development of Id2- and Id3-deficient lymphomas

To gain insight into the mechanism that underpins the development of lymphoma in *Id2-* and *Id3-*deficient mice, transcription signatures were analyzed from a set of lymphomas using RNA-seq. We found that *Id2-* and *Id3-*deficient lymphomas revealed transcription profiles that overlapped with those derived from *Id2-* and *Id3-*deficient CD4SP cells (Fig. 7F). Specifically, ∼2100 genes were differentially expressed in *Id2-* and *Id3-*deficient lymphomas versus *Id2*- and *Id3*-depleted CD4SP cells (Supplemental Fig. 12A,B). The majority of isolated lymphomas expressed a limited TCR repertoire, arguing against polyclonal expansion (Supplemental Fig. 12C,D). We found that the differences in transcription profiles between lymphomas and CD4SP cells were closely associated with genes involved in metabolism, proliferation, and the immune response (Supplemental Fig. 12E). As predicted, we found substantial decreased levels of *Foxo1* and *Foxo3* as well as an elevated abundance of *c-Myc* and *Hif1a* (Fig. 7G). Conspicuous among the spectrum of aberrantly expressed genes was the *Cdkn2a* locus. Notably, *Cdkn2a* transcript abundance was high in *Id2*^fl/fl^*Id3*^fl/fl^*IL7R*^Cre^ CD4SP cells but low in lymphoma cells (Fig. 7G). The *Cdkn2a* locus encodes for two tumor suppressors: *p16Ink4a* and *p19Arf*. *p19Arf* is activated by *c-Myc* and has been demonstrated to suppress lymphomagenesis (Kamijo et al. 1997; Zindy et al. 2003). We found that *p19Arf* but not *p16Ink4a* transcript abundance was elevated in the self-renewing innate variant T_FH_-like population (Fig. 7H). In contrast, *p19Ar*f transcript levels were virtually undetectable in Id-deficient lymphomas (Fig. 7H). Taken together, these data point to a regulatory circuitry that maintains thymocyte quiescence and provide a mechanism involving *c-myc* and *p19Arf* that underpins the development of T-cell lymphoma in *Id2-* and *Id3-*deficient T cells.

## Discussion

Previous studies have demonstrated that positive selection is enforced by the E and Id protein module (Bain et al. 1999; Jones and Zhuang 2007; Jones-Mason et al. 2012). Here we examined how the Id proteins orchestrate thymocyte selection. Based on this analysis, we propose two key steps that involve Id2 and Id3 during the positive selection process. The first step involves the induction of *Id3* expression by TCR-mediated signaling. The second step involves the induction of *Id2* expression in cells that have already received a TCR signal. We demonstrate here that *Id2*- and *Id3*-depleted thymocytes were not able to pass this second step of selection, with the exception of a slowly expanding population of cells that was characterized by an innate-like transcription signature. Innate T cells, including iNKT, T-CD4T, and MAIT cells, are selected by a mechanism that is distinct from that of conventional CD4SP thymocytes (Li et al. 2005; Lee et al. 2010; Alonzo and Sant’Angelo 2011; Gold and Lewinsohn 2013). Specifically, iNKT and MAIT cells are MHC class I-like-restricted, involving innate TCR signaling. Additionally, iNKT and T-CD4 T cells are selected by DP thymocytes rather than by thymic epithelial cells. Hence, we propose that differences in TCR-mediated signaling—conventional or innate-mediated TCR signaling—affect the duration and/or periodicity of *Id3* and/or *Id2* transcription and that CD4 T cells selected by MHC class II in thymic epithelial cells but not innate CD4 T cells require *Id2* and *Id3* expression. Differences in the strength, periodicity, and timing of *Id2* and *Id3* expression would then instruct progenitors to commit to either the adaptive or innate T-cell lineage.

We found that *Id2*- and *Id3*-depleted positively selected thymocytes express a T_FH_-like program of gene expression. Particularly intriguing was the decline of *Foxo1* and *Foxp1* abundance in *Id*-depleted CD4SP thymocytes. *Foxo1* and *Foxp1* are well-characterized suppressors of the T_FH_ cell fate (Hedrick et al. 2012; Wang et al. 2014; Xiao et al. 2014). These data bring into question how Id proteins and *Foxo1* and *Foxp1* are linked. We found E2A-bound sites across regulatory regions associated with the *Foxo1* and *Foxp1* loci. Hence, we suggest that in CD4SP thymocytes, the E proteins act as transcriptional repressors that interfere with *Foxo1* and *Foxp1* transcription. High levels of *Id2* and *Id3* antagonize the DNA-binding activity of E proteins, relieving *Foxo1* and *Foxp1* from E2A/HEB-mediated repression. Elevated levels of *Foxo1* and *Foxp1* in turn would prevent the premature activation of a T_FH_ lineage-specific program of gene expression (Supplemental Fig. 13A).

A notable feature of our findings is the development of an innate variant T_FH_ cell population in mice depleted for *Id2* and *Id3* expression. Are these cells genuine T_FH_ cells? We noted overlap but also significant differences in transcription signatures upon comparing T_FH_ and *Id*-depleted innate T_FH_ cells. Notably, *Bcl6* and *Ascl2* expression, closely associated with a T_FH_-specific program of gene expression (Crotty 2014), were not modulated, at least transcriptionally, in *Id*-depleted CD4SP thymocytes. Despite these differences, we found that *Id*-depleted innate variant T_FH_ cells secreted high levels of IL-4, required interaction with MHC class I-like molecules, and their presence was closely associated with the spontaneous development of GCs. Hence, we suggest that these cells represent a distinct subset of T_FH_ cells (Supplemental Fig. 13B).

A key aspect of the findings reported here involves a slowly expanding population of innate T_FH_-like cells. How do Id2 and Id3 regulate thymocyte quiescence? We found that multiple pathways known to maintain lymphocyte quiescence were affected by depletion of *Id2* and *Id3* expression. Prominent among these was the FOXO–mTOR module. The roles for the FoxO proteins in maintaining thymocyte quiescence and acting as tumor suppressors are well documented (Paik et al. 2007; Kerdiles et al. 2009; Hedrick et al. 2012). Similarly, spontaneous activation of the mTORC1 pathway in *Tsc1*-deficient T cells leads to an increase in cell size and loss of quiescence, phenotypes that are equivalent to that described here for *Id2*- and *Id3*-depleted CD4SP thymocytes (Yang et al. 2011b). As mentioned previously, the entire ensemble of *Foxo* loci, including *Foxo1*, *Foxo3*, and *Foxo4*, appears to be controlled by the E*–*Id protein module. Likewise, the AKT–FOXO–mTOR pathway was activated in *Id2*- and *Id3*-depleted CD4SP thymocytes as well as in *Id3*-deficient innate γδ T cells. Multiple levels of regulation by the E*–*Id axis appear to be involved here: (1) modulation of Foxo transcript levels, (2) activation of the AKT pathway plausibly mediated by changes in the expression of an ensemble of PTPN phosphatases that are modulated upon depletion of Id2 and Id3, and (3) activation of the mTORC pathway in part by induction of Rps6ka2 expression. We suggest that this form of regulation is not restricted to innate variant T_FH_ and a subset of Vγ1.1 γδ T cells. Rather, we propose that the regulation of the PI3K–FOXO–mTOR pathway by the E*–*Id axis is a general mechanism that underpins the homeostasis of lymphoid progenitors and self-renewing committed B and T lymphoid cells.

Finally, we found that *c-Myc* and *p19Arf* levels were elevated in *Id*-depleted CD4SP thymocytes. *p19Arf* is a well-known tumor suppressor that is regulated by c-Myc (Lowe and Sherr 2003). Previous studies have demonstrated that c-myc expression is directly regulated by E proteins (Schwartz et al. 2006). Hence, we suggest that loss of Id expression leads to elevated c-Myc abundance, which in turn leads to the induction of *p19Arf* expression. We speculate that cellular expansion upon depletion of *Id2* and *Id3* expression is attenuated by the induction of *p19Arf* expression. This then would lead to a population that slowly self-renews. How do lymphomas develop in *Id2*- and *Id3*-depleted thymocytes? We speculate that, through mechanisms yet to be determined, *p19Arf* expression is inactivated in a single progenitor, releasing the brakes and ultimately leading to the development of a monoclonal αβ T-cell lymphoma (Supplemental Fig. 13C; von Boehmer 2004).

The mechanisms that underpin the development of αβ T-cell lymphomas in *Id2*- and *Id3*-depleted mice overlap with those observed in human Burkitt lymphoma and a murine model of Burkitt lymphoma (Sander et al. 2012; Schmitz et a. 2012). It is now established that the development of Burkitt lymphoma is closely associated with high levels of *c-Myc* expression and mutations localized across the HLH region of *Id3* (Love et al. 2012; Schmitz et al. 2012). Likewise, we found that *Id2*- and *Id3*-depleted murine T-cell lymphomas expressed relatively high levels of *c-Myc* expression. There are also similarities between the two sets of lymphomas as they relate to the PI3K pathway. Burkitt lymphomas are associated with increased PI3K signaling and display an activated AKT pathway (Sander et al. 2012; Schmitz et al. 2012). We found that Id-deficient CD4 T cells displayed decreased abundance of *Foxo1/3* expression as well as activated AKT and mTORC1 pathway. Upon examining transcription signatures in human T-cell lymphomas, we found that changes in *Id2*, *Foxo1*, and *Foxo3* abundance were significantly associated with the development of human T-cell lymphoma, (Supplemental Fig. 14; Piccaluga et al. 2007). Finally, since a very high fraction of aged mice display symptoms of inflammatory disease, it is conceivable that chronic inflammation contributes to the development of T-cell lymphoma in *Id2*- and *Id3*-deficient mice similarly to as described for viral infections associated with the development of human lymphomas.

## Materials and methods

### Mice

C57BL/6, *Id3*^f/f^, *Id2*^f/f^, *Id3*^−/−^, *Id2*^YFP/+^, *Id3*^GFP/+^, *IL7Rα*^Cre^, *CD4*^Cre^, *dLck*^Cre^, and *ROSA*^YFP/YFP^ mice were bred and housed in specific pathogen-free conditions in accordance with the Institutional Animal Care and Use Guidelines of the University of California at San Diego.

### Flow cytometry and cell sorting

Single-cell suspensions from the bone marrow, thymus, and spleen were stained with the following: FITC-labeled, PE-labeled, APC-labeled, APC-Cy7-labeled, Pacific Blue-labeled, Alexa Fluor 700-labeled, Alexa Fluor 780-labeled, PerCP-Cy5.5-labeled, PE-Cy7-labeled, or biotin-labeled monoclonal antibodies, which were purchased from BD PharMingen, including CD11c (HL3), CD44 (IM7), CXCR5 (2G8), IgG1 (A85-1), CD95 (Jo-2), CD138 (281-2), GL-7, Ki67 (B56), and phospho-AKT (M89-61). CD8 (53-6.7), CD19 (ID3), CD38 (90), CD62L (MEL-14), CD44 (IM7), CD69 (H1.2F3), CD127 (A7R34), B220 (RA3-6B2), Mac1 (M1/70), Gr1 (RB6-8C5), Nk1.1 (PK136), Ter119 (TER119), TCRβ (H57), TCRγδ (GL3), PD-1 (J43), ICOS (7E.17G9), IgD (11-26), CCR7 (4B12), CXCR4 (2B11), TBR2 (Dan11mag), phosph-S6 (supk43k), phosph-4E-BP1 (V3NTY24), and AnnexinV were purchased from eBioscience. FceR1 (MAR-1), CD3ε (2C11), CD4 (GK1.5), CD8 (53-6.7), CD11b (M1/70), CD24 (M169), CD25 (PC61), PD-1 (RMP1-30), and F4/80 (BM8) were obtained from Biolegend. CCR9 was purchased from R&D Systems. PLZF (clone D9) was obtained from Santa Cruz Biotechnology. PE-conjugated CD1d tetramer was generously provided by the National Institutes of Health Tetramer Core Facility (mCD1d/PBS57). Biotinylated antibodies were labeled with streptavidin-conjugated Qdot-665, or Qdot-605 (Invitrogen). Clone 2.4 G2 anti-CD32:CD16 (eBioscience) was used to block FcRs. Dead cells were removed from analysis and sorting by staining with propidium iodide (PI) (Sigma-Aldrich). BrdU incorporation was performed using a BD BrdU flow kit. Annexin V staining was performed using an eBioscience Annexin V apoptosis detection kit APC. Treg cell staining kit (eBioscience) was used for intracellular staining of PLZF and Foxp3 and Eomes detection. Intracellular staining kit (eBioscience) was used for phosph-S6/4E-BP1 staining. BD Cytofix fixation buffer and BD Phosflow perm buffer III were used for phospho-AKT staining. Samples were collected on a LSRII (BD Biosciences) and were analyzed with FlowJo software (Tree Star). Cells were sorted on a FACSAria. For intracellular staining of IFN-γ and IL-4, MACS-purified CD4SP and DN TCRβ^+^ cells from thymi were cultured in the presence of phorbol 12-myristate 13-acetate (PMA) plus ionomycin (4 h) and Golgi stop (2 h). After culture, cells were stained with anti-CD4, CD8, TCRβ, and CD3ε antibodies. Intracellular staining was performed with the BD Biosciences Cytofix/Cytopermkit.

### RNA-seq analysis

Total RNA and the library preparations were described previously (Miyazaki et al. 2014). The strand-specific RNA-seq libraries were sequenced with a HiSeq 2500 sequencer (Illumina). Alignment and trimming of reads were performed using the OSA algorithm against the mm10 murine genome reference in Arraystudio (Omicsoft) (Hu et al. 2012). RNA transcripts were quantified using RSEM methods (http://deweylab.biostat.wisc.edu/rsem) as implemented in Arraystudio (Omicsoft). Abundance values (counts) were normalized and compared with calculated *P*-values using DESeq (http://www-huber.embl.de/users/anders/DESeq). Genes whose abundance values were <10 in all samples were removed. MultiExperiment Viewer software was used to generate heat maps and for hierarchical clustering. GO analyses and visualization files were generated using HOMER (http://biowhat.ucsd.edu/homer), and read pile-ups were visualized using the University of California at Santa Cruz Genome Browser.

### Histology

Tissues were fixed in 4% paraformaldehyde (Electron Microscopy Sciences). Fixed tissues were embedded in paraffin and sliced, followed by haematoxylin and eosin (H&E) staining.

### ELISPOT

Cells were cultured overnight at 37°C on 96-well MultiScreen-HA filter plates (Millipore) precoated with goat anti-murine Ig(H+L) capture antibodies (Southern Biotechnology Associates [SBA]). Spots were visualized with goat anti-murine IgM or IgG1 antibodies conjugated to HRP, and color was developed by 3-amino-9-ethyl carbazole (Sigma-Aldrich).

### Statistical analyses

*P*-values were calculated with the two-tailed Student’s test for two-group comparison, as applicable, with Microsoft Excel software.

### Data access

RNA-seq data have been deposited at Gene Expression Omnibus under accession number GSE64779.

## Supplementary Material

Supplemental Material
