# Supplementary material for: The E–Id protein axis modulates the activities of the PI3K–AKT–mTORC1–Hif1a and c-myc/p19Arf pathways to suppress innate variant TFH cell development, thymocyte expansion, and lymphomagenesis
Source: Genes Dev. 2015 Feb 15;29(4):409–25. doi: 10.1101/gad.255331.114 (PMC4335296; doi:10.1101/gad.255331.114)
Supplement: Supplemental Material [file supp_29_4_409__index.html]

Supplemental Material 

# The E–Id protein axis modulates the activities of the PI3K–AKT–mTORC1–Hif1a and c-myc/p19Arf pathways to suppress innate variant TFH cell development, thymocyte expansion, and lymphomagenesis

## Supplemental Material

**Files in this Data Supplement:**

- Supp Figures.pdf
