# Supplementary material for: The E–Id protein axis modulates the activities of the PI3K–AKT–mTORC1–Hif1a and c-myc/p19Arf pathways to suppress innate variant TFH cell development, thymocyte expansion, and lymphomagenesis
Source: Genes Dev. 2015 Feb 15;29(4):409–25. doi: 10.1101/gad.255331.114 (PMC4335296; doi:10.1101/gad.255331.114)
Supplement: Supplemental Material [file supp_29.4.409_SuppFigs.pdf]

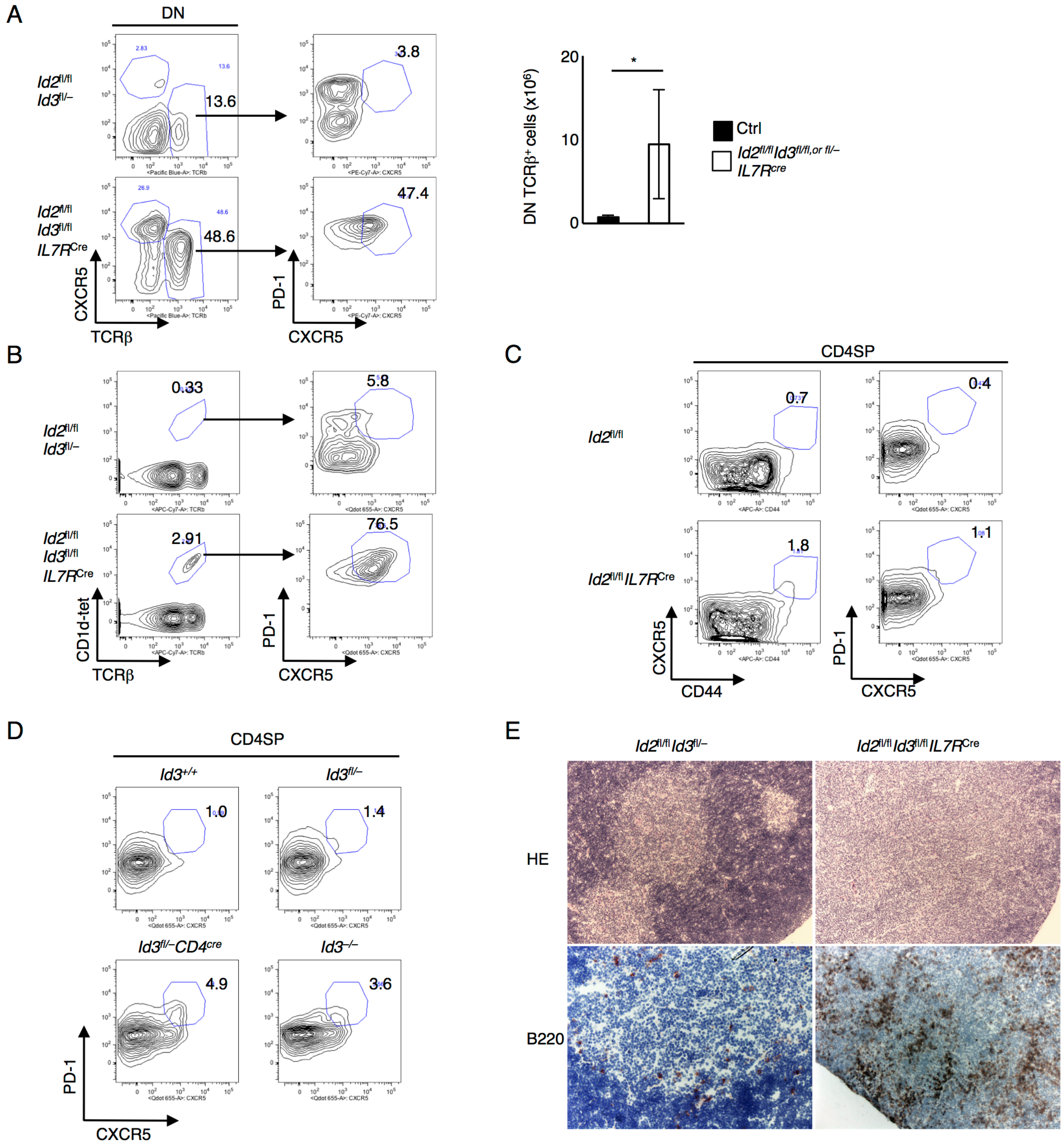

Figure S1. Related to Figure 1.The development of innate T_{FH}-like cells in thymi derived from *Id2*$^{fl/fl}$*Id3*$^{fl/fl}$ *IL7R*^{Cre} mice. (A) Flow cytometric analysis of CXCR5 versus TCRβ expression gated on $CD4^{-}CD8^{-}$ (DN) thymocytes, and CXCR5 versus PD-1 expression gated on DN;$TCR\beta^{+}$ cells derived from 5-week-old *Id2*$^{fl/fl}$ *Id3*$^{fl/fl}$*IL7R*^{Cre} thymus. Graph shows absolute numbers of DN;$TCR\beta^{+}$cells. ★, $P < 0.05$ (Student's t test). (B) Flow cytometric analysis of TCRβ versus CD1d-tet expression in total thymocytes, and CXCR5 versus PD-1 expression gated on CD1d-tet^{+}TCRβ^{+} iNKT cells. (C) Flow cytometric analysis of CXCR5 versus CD44 expression, and CXCR5 versus PD-1 expression, gated on CD4SP cells derived from *Id2*$^{fl/fl}$*IL7R*^{Cre} thymi. (D) CXCR5 versus PD-1 expression gated on CD4SP cells derived from control (*Id3*$^{+/+}$ and *Id3*$^{fl/-}$), *Id3*$^{fl/-}$ CD4^{Cre}, and *Id3*$^{-/-}$ mice. (E) Representative hematoxylin-eosin staining (HE; top) and immunostaining directed against B220 (bottom) from 5-week-old *Id2*$^{fl/fl}$*Id3*$^{fl/fl}$*IL7R*^{Cre} or littermate control mice. Original magnification: x100.

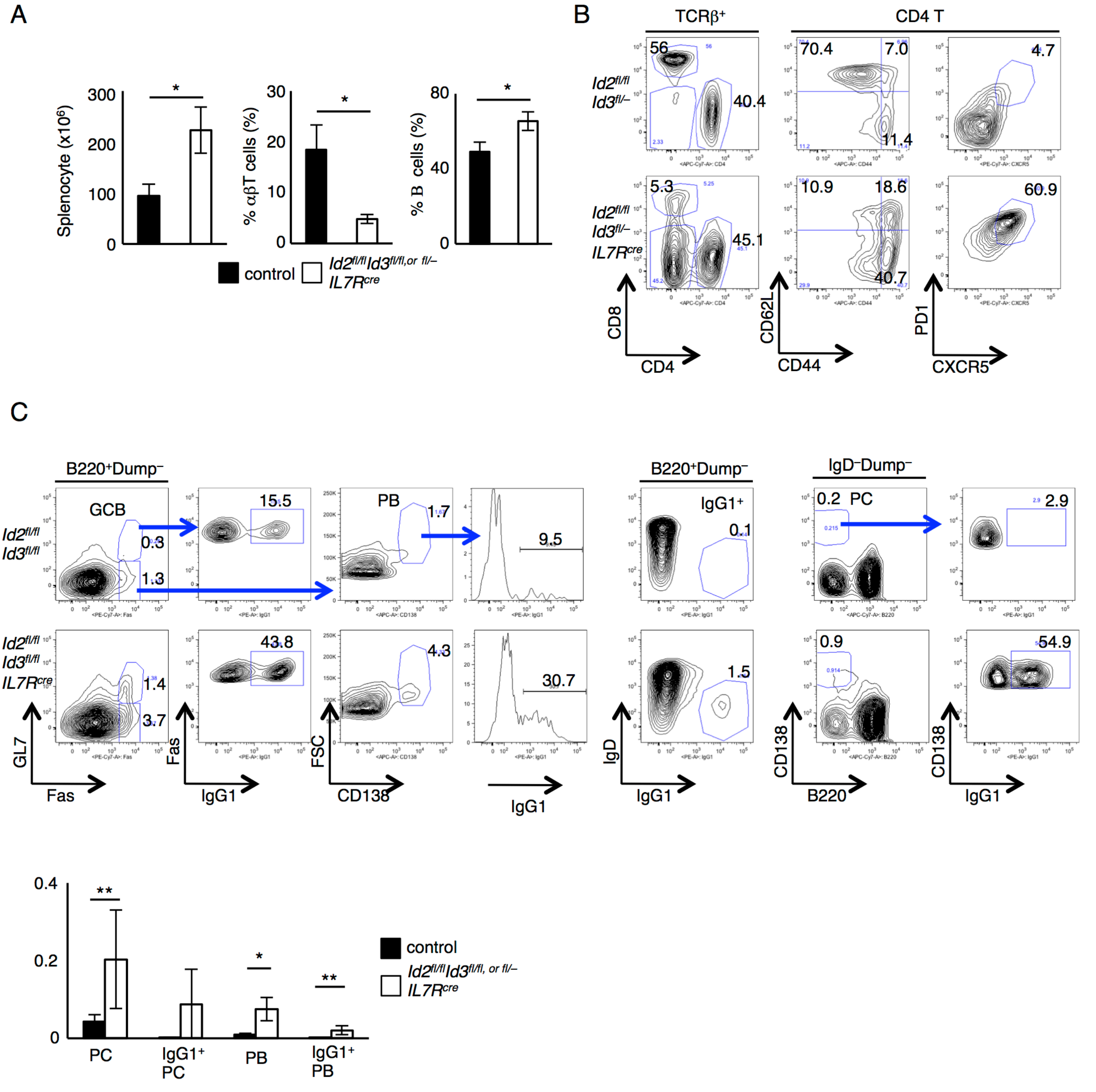

Figure S2. Related to Figure 1. T_{FH} cells, and IgG1-class switched and germinal center B cellls in the spleen derived from 5-week-old *Id2*$^{fl/fl}$*Id3*$^{fl/fl}$*IL7R*^{Cre} mice. (A) Absolute number of splenocytes and frequency of αβT cells and B cells in spleen. (B) Flow cytometric analysis of CD4 versus CD8 gated on TCRβ^{+} T cells, and CD44 versus CD62L expression and CXCR5 versus PD-1 expression, gated on CD4 T cells in the spleen. (C) Flow cytometric analysis of GC B cells (Fas^{+}GL7^{+}), IgG1-class switched GC B cells, Plasma blast cells (PB)(Fas^{+}GL7^{-}CD138^{+}large cell), IgG1-class switched PB, IgG1 class switched B cells (IgG1^{+}) (IgG1^{+}IgD^{-}), Plasma cell (PC) (IgD^{-}Dump^{-}B220^{-}CD138^{+}), and IgG1-class switched PC. ★, *P* < 0.05, ★★, *P* <0.01 (Student's *t* test).

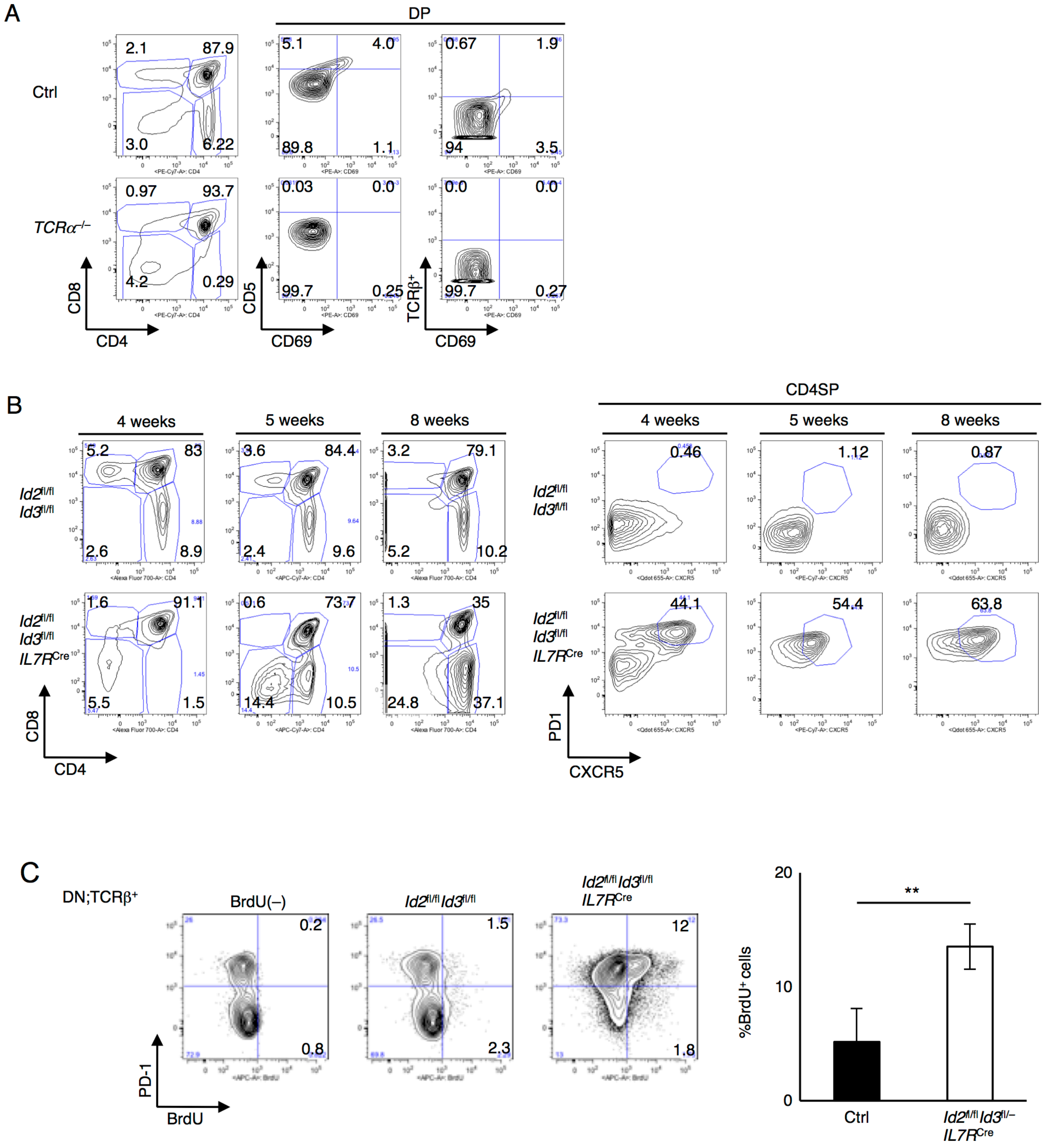
A
DP
Ctrl
TCRα−/−
2.1
87.9
3.0
6.22
0.97
93.7
4.2
0.29
5.1
4.0
89.8
1.1
0.03
0.0
99.7
0.25
0.67
1.9
94
3.5
0.0
0.0
99.7
0.27
CD8
CD4
CD5
CD69
TCRβ+
CD69
B
CD4SP
4 weeks
5 weeks
8 weeks
4 weeks
5 weeks
8 weeks
Id2fl/fl Id3fl/fl
Id2fl/fl Id3fl/fl IL7RCre
5.2
83
2.6
8.9
3.6
84.4
2.4
9.6
3.2
79.1
5.2
10.2
1.6
91.1
5.5
1.5
0.6
73.7
14.4
10.5
1.3
35
24.8
37.1
CD8
CD4
0.46
1.12
0.87
44.1
54.4
63.8
PD1
CXCR5
C
DN;TCRβ+
BrdU(–)
Id2fl/fl Id3fl/fl
Id2fl/fl Id3fl/fl IL7RCre
0.2
0.8
1.5
2.3
12
1.8
PD-1
BrdU
20
10
0
%BrdU+ cells
**
Ctrl
Id2fl/fl Id3fl/– IL7RCre

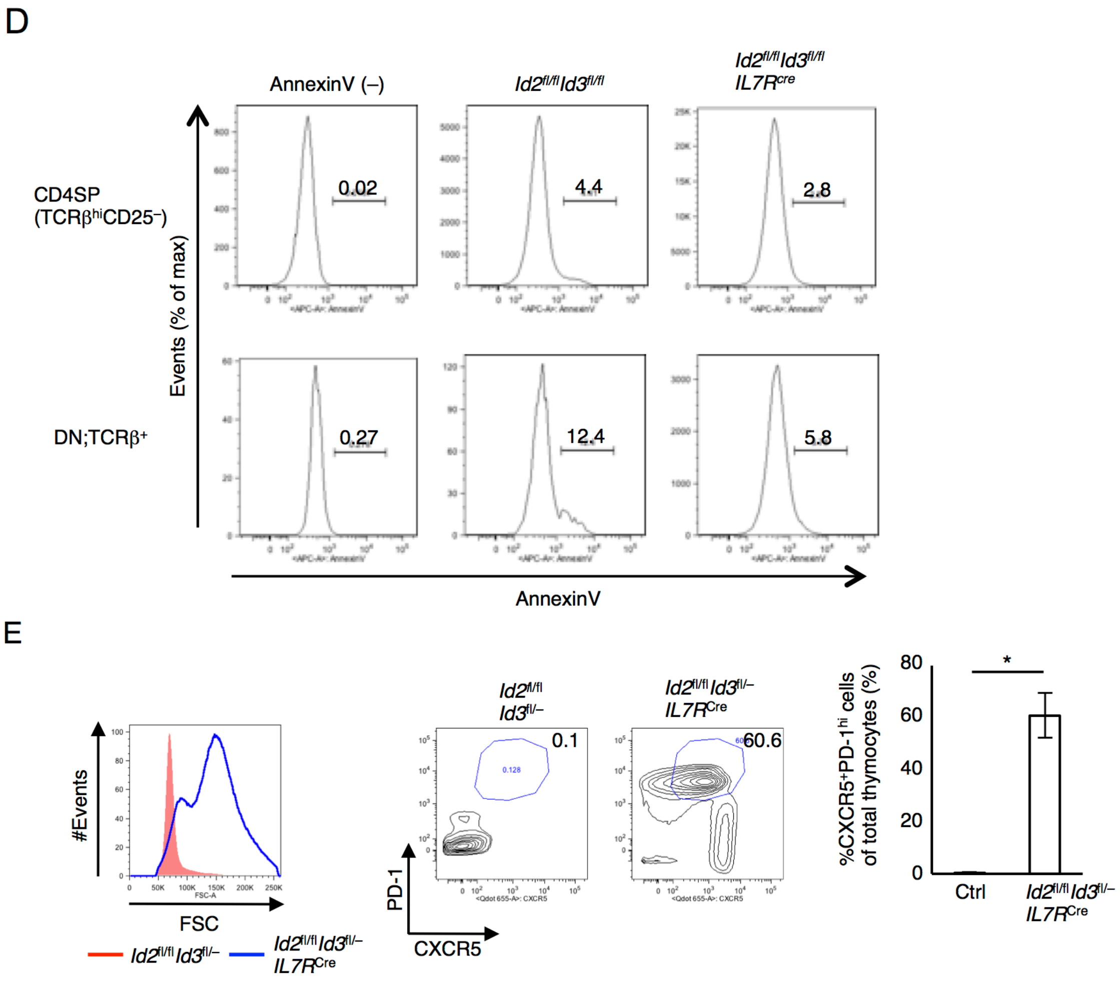

Figure S3. Related to Figure 3. Expansion and increased cell size of CXCR5$^+$PD-1$^+$ CD4SP cells in *Id2*$^{fl/fl}$ *Id3*^{flfl}*IL7R*^{Cre} thymi. (A) Flow cytometric analysis of CD4 versus CD8 expression gated on total thymocytes, CD69 versus CD5 expression and CD69 versus TCRβ expression, gated on CD4$^+$CD8$^+$ DP cells, derived from TCRα$^{-/-}$ thymus. (B) Expression of CD4 versus CD8 in total thymocytes, and CXCR5 versus PD-1 gated on CD4SP cells, derived from 4-, 5-, and 8-week-old control or *Id2*$^{fl/fl}$*Id3*$^{fl/fl}$*IL7R*^{Cre} mice. (C) BrdU incorporation in DN;TCRβ$^+$ cells derived from *Id2*$^{fl/fl}$*Id3*$^{fl/fl}$ *IL7R*^{Cre} and *Id2*$^{fl/fl}$*Id3*$^{fl/fl}$ mice. Numbers adjacent in quadrants indicate percentages of cells in each. Lower panel indicates frequency of cells that incorporate BrdU. Data represent the mean ± SD from four mice. **, P <0.01 (Student's *t* test). Data are representative from three independent experiments with three or four 8-10 week-old mice each. (D) AnnexinV staining in CD4SP and DN;TCRβ$^+$ cells. Numbers above lines indicate fraction of AnnexinV$^+$ cells. (E) Flow cytometric analysis of cell size and CXCR5 versus PD-1 expression gated on total thymocytes derived from 6-month-old littermate or *Id2*$^{fl/fl}$*Id3*$^{fl/fl}$*IL7R*^{Cre} mice. Graph shows the frequency of CXCR5$^+$PD-1$^+$ cells of total thymocytes in 6-month-old *Id2*$^{fl/fl}$*Id3*$^{fl/fl}$*IL7R*^{Cre} mice. Data represent the mean ± SD from three mice. ★, P < 0.05, (Student's *t* test).

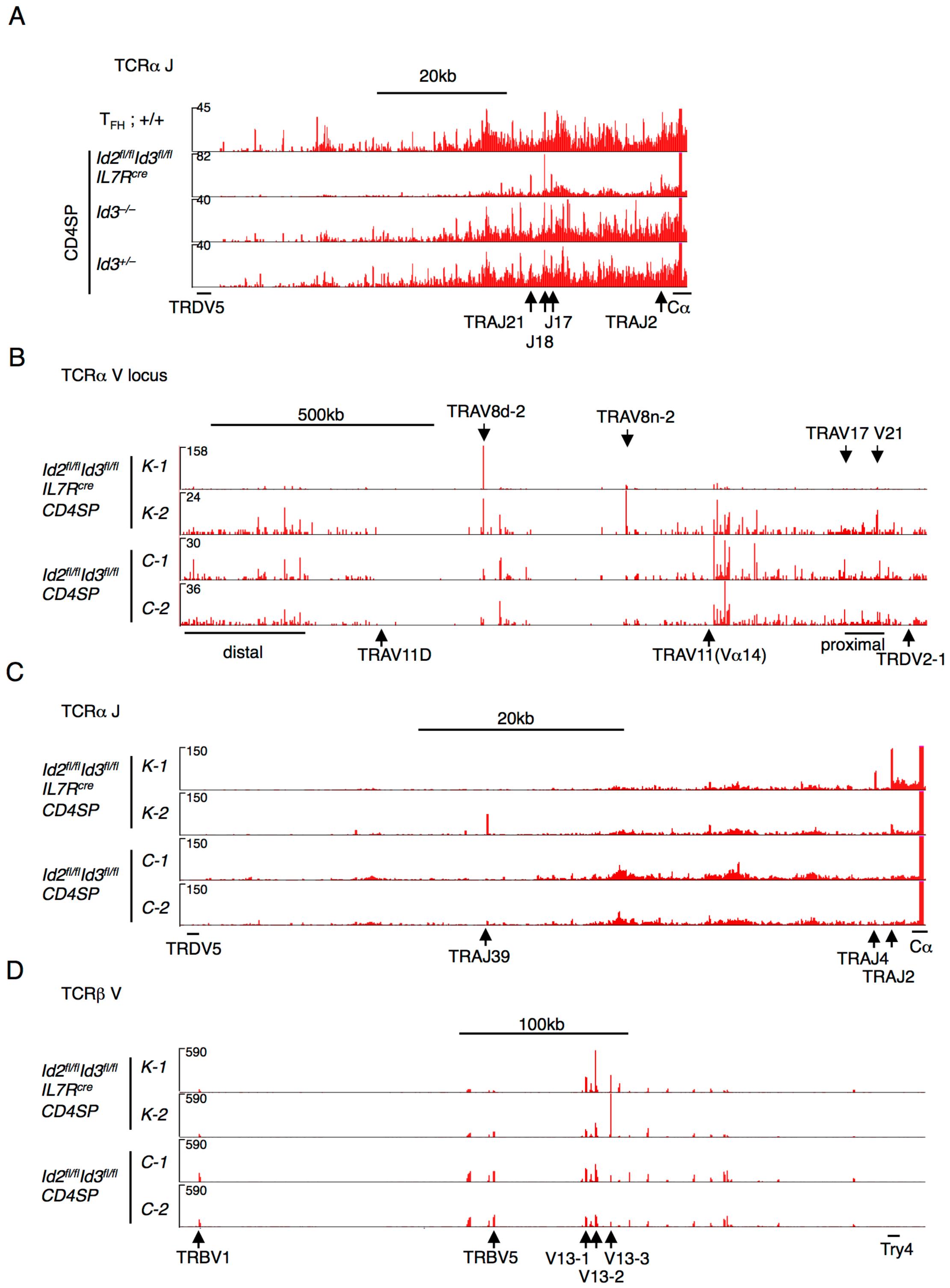

Figure S4. Related to Figure 3. Highly restricted TCRα and TCRβ repertoire in CD4SP cells isolated from *Id2*$^{fl/fl}$*Id3*$^{fl/ll}$IL7R^{cre} mice. (A) RNA-Seq analysis across the TCR Jα region, as seen in Figure 3G,H.. (B) RNA-seq analysis across the TCR Vα region. (C) RNA-Seq analysis across the TCR Jα region. (D) RNA-Seq analysis across theTCRβ V region. (B-D) RNA was isolated from CD4SP cells derived from *Id2*$^{fl/fl}$*Id3*$^{fl/fl}$*IL7R*^{Cre} (K-1, K-2) and control (C-1, C-2) mice.

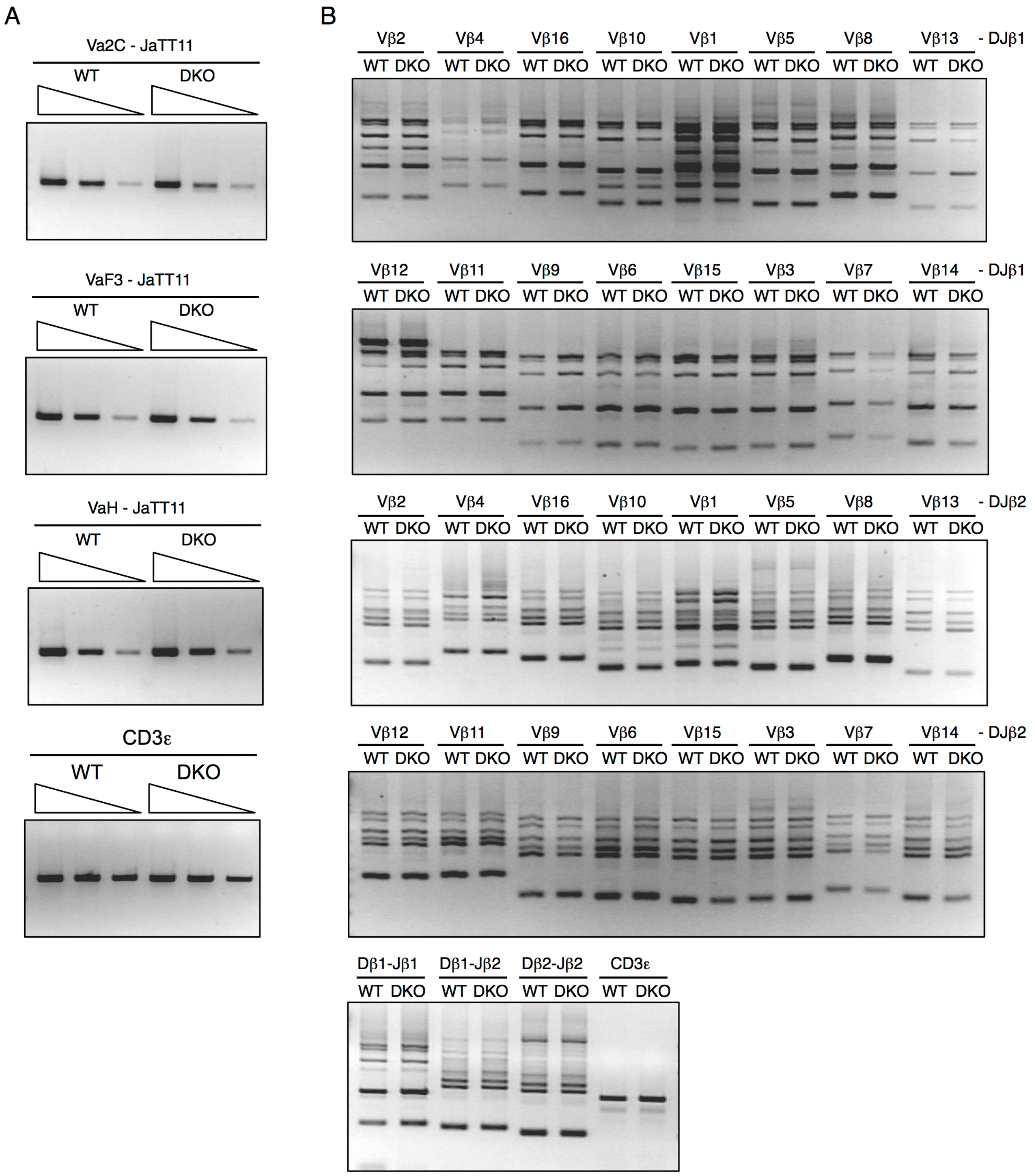

Figure S5. Related to Figure 3. *Id2*- and *Id3*-deficient DP thymocytes lack defects in TCRα and TCRβ gene rearrangements.

(A) PCR analysis involving TCRα rearrangements in DP thymocytes. Four-fold serially diluted genomic DNAs prepared from control or $Id2^{fl/fl}Id3^{fl/fl}IL7R^{Cre}$ DP thymocytes were analyzed for Vα-Jα rearrangements by PCR using Vα2C, VαF3 or VαH upstream primers in conjunction with JαTT11 downstream primes. Equal DNA quantities were verified by PCR of the *Cd3e* gene. PCR products were visualized by ethidium bromide gel staining. Data are representative of two independent experiments. (B) PCR analysis of TCRα rearrangement in DP thymocytes. Genomic DNAs were analyzed for Vβ-DJβ1, Vβ-DJβ2, Dβ1-Jβ1, Dβ1-Jβ2 and Dβ2-Jβ2 rearrangements by PCR using Vβ or Dβ upstream primers in conjunction with Jβ1 or Jβ2 downstream primers.

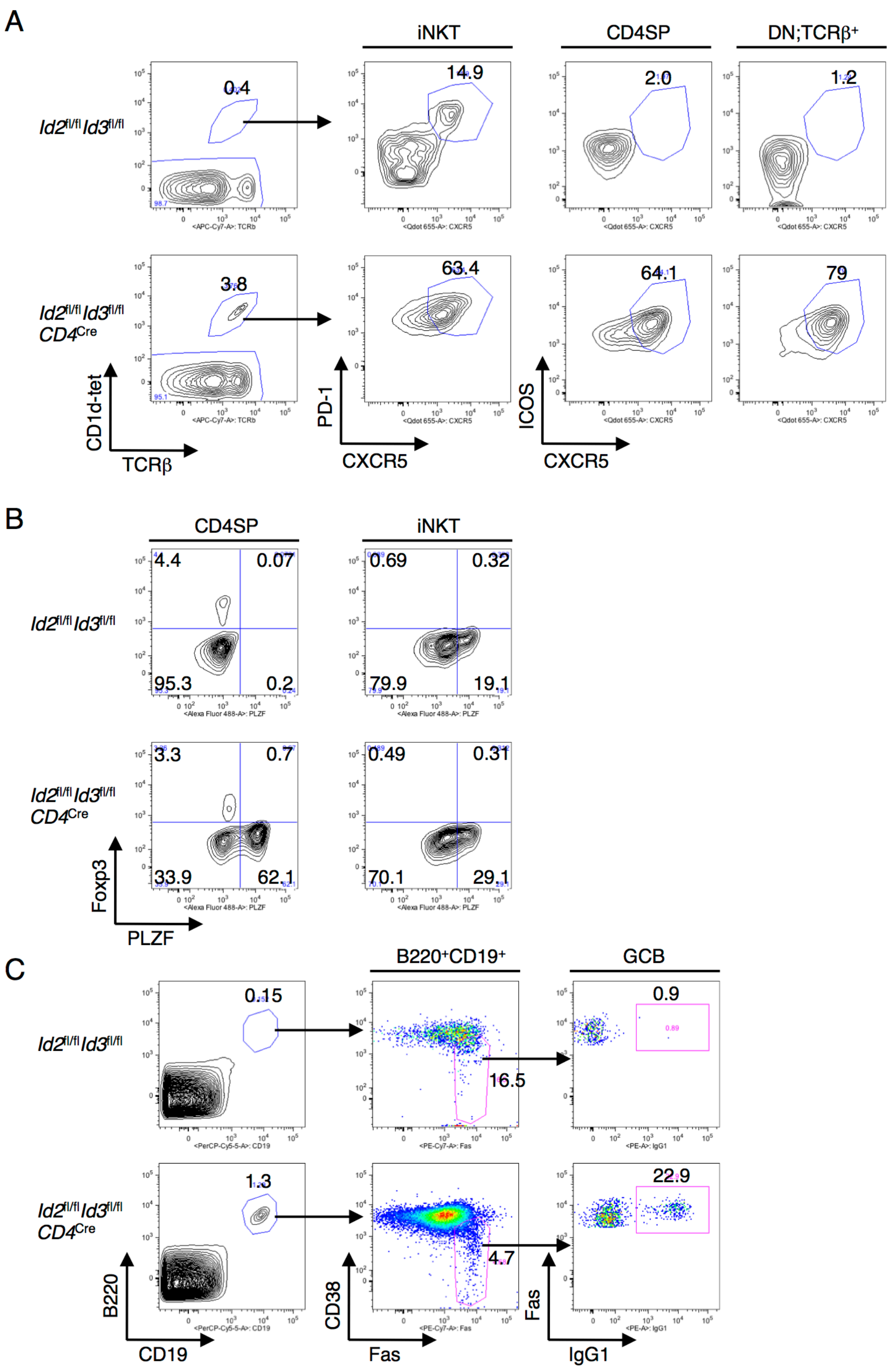

Figure S6. Related to Figure 4. Innate T_{FH}-like cells, $iNKT_{FH}$ cells and increased numbers of thymic B cells in *Id2*$^{fl/fl}$*Id3*$^{fl/fl}$*CD4*^{Cre} thymi.
(A) Flow cytometric analysis of TCRβ versus CD1d-tet expression in total thymocytes, CXCR5 versus PD-1 expression gated on CD1d-tet^{+}TCRβ^{+} iNKT cells, CXCR5 versus ICOS expression gated on CD4SP or DN;TCRβ^{+} cells. (B) Flow cytometric analysis of PLZF versus Foxp3 expression gated on CD4SP cells and iNKT cells. (C) The expression of CD19 and B220, Fas and CD38 gated on CD19^{+}B220^{+} thymic B cells and IgG1 and Fas gated on Fas^{+}CD38^{lo} cells.

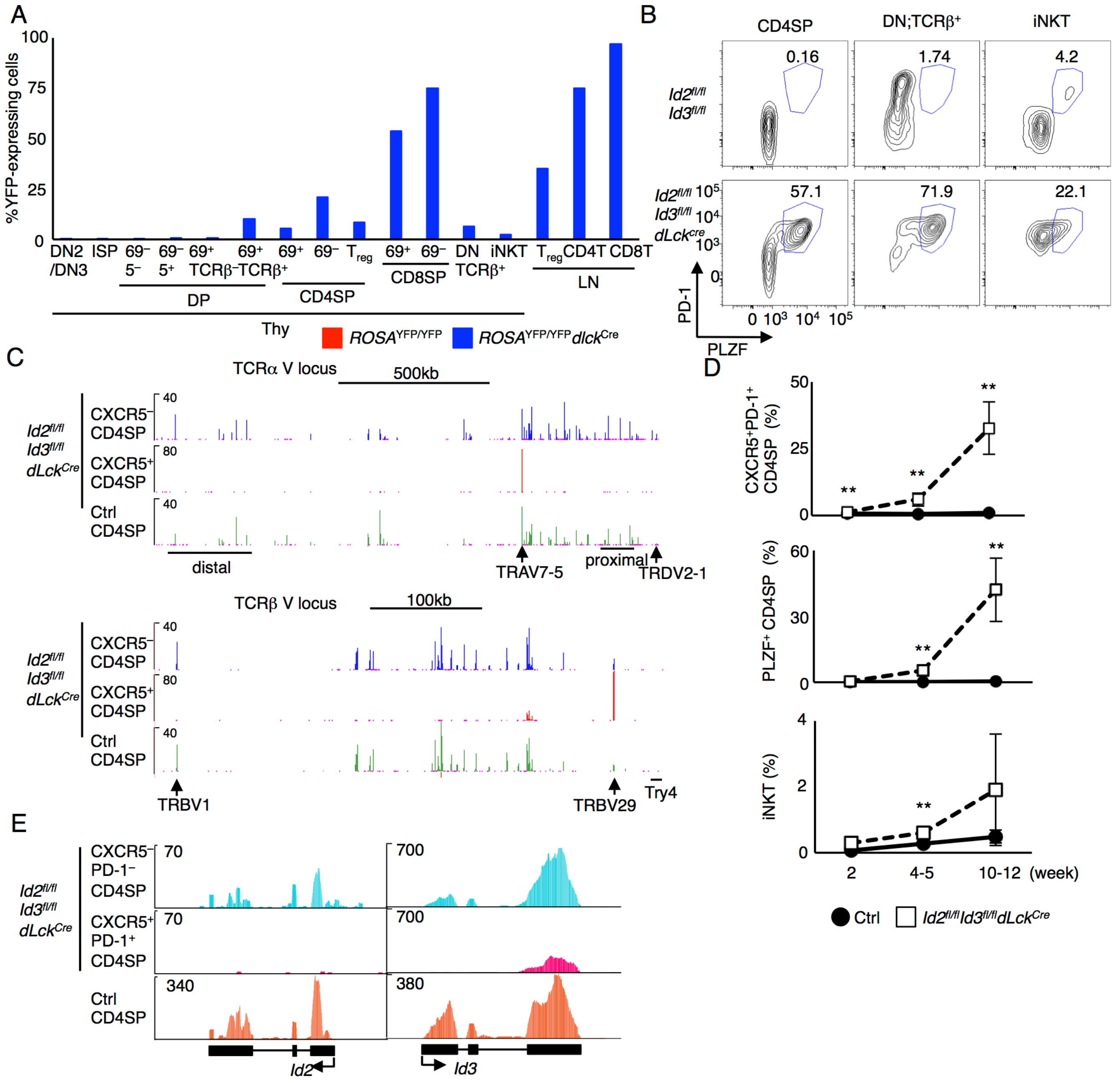

Figure S7. Related to Figure 4. *Id2* and *Id3* deletion beyond the TCR checkpoint results in the expression of CXCR5 and expansion of innate T cells. (A) Percentages of YFP-expressing cells at various stages of thymocyte development in *ROSA*$^{YFP/YFP}$*dLck*^{Cre} thymus. (B) Flow cytometric analysis of PLZF and PD-1 expression, gated on CD4SP (CD4$^+$CD8$^-$TCRβ$^+$CD1d-tet$^-$), DN;TCRβ$^+$ (CD4$^-$CD8$^-$TCRβ^{hi}CD1d-tet$^-$), and iNKT cells, derived from 10-week-old control or *Id2*$^{fl/fl}$*Id3*$^{fl/fl}$*dLck*^{Cre} mouse. (C) RNA-seq analysis for TCR Vα and TCR Vβ regions in wild-type and *Id2*- and *Id3*-depleted CD4SP thymocytes. mRNA was isolated from sorted CXCR5$^+$PD-1$^+$ CD4SP cells (middle) and CXCR5$^-$PD-1$^-$ CD4SP cells (top) from *Id2*$^{fl/fl}$*Id3*$^{fl/fl}$ *dLck*^{Cre} mouse and CD4SP cells (Ctrl; bottom) from control mouse. Numbers of reads are indicated for each of the tracks. One experiment was performed. (D) Indicated are the frequencies of CXCR5$^+$PD-1$^+$ or PLZF$^+$ cells in CD4SP cells, and iNKT cells in total thymocytes with aging. Data were derived from four independent experiments (mean ± s.d.; n = five (2-week), six (4-5-week), seven (10-12week) biological replicates). ★★, P <0.01 (Student's t test). (E) RNA-seq analysis across *Id2* and *Id3* loci, presented in reads per million reads aligned. RNA were isolated from sorted CXCR5$^-$PD-1$^-$ or CXCR5$^+$PD-1$^+$ CD4SP (CD4$^+$CD8$^-$TCRβ$^+$CD1d-tet$^-$) cells from identical *Id2*$^{fl/fl}$*Id3*$^{fl/fl}$*dLck*^{Cre} mice. Arrows indicate transcriptional start site and direction of transcription.

A

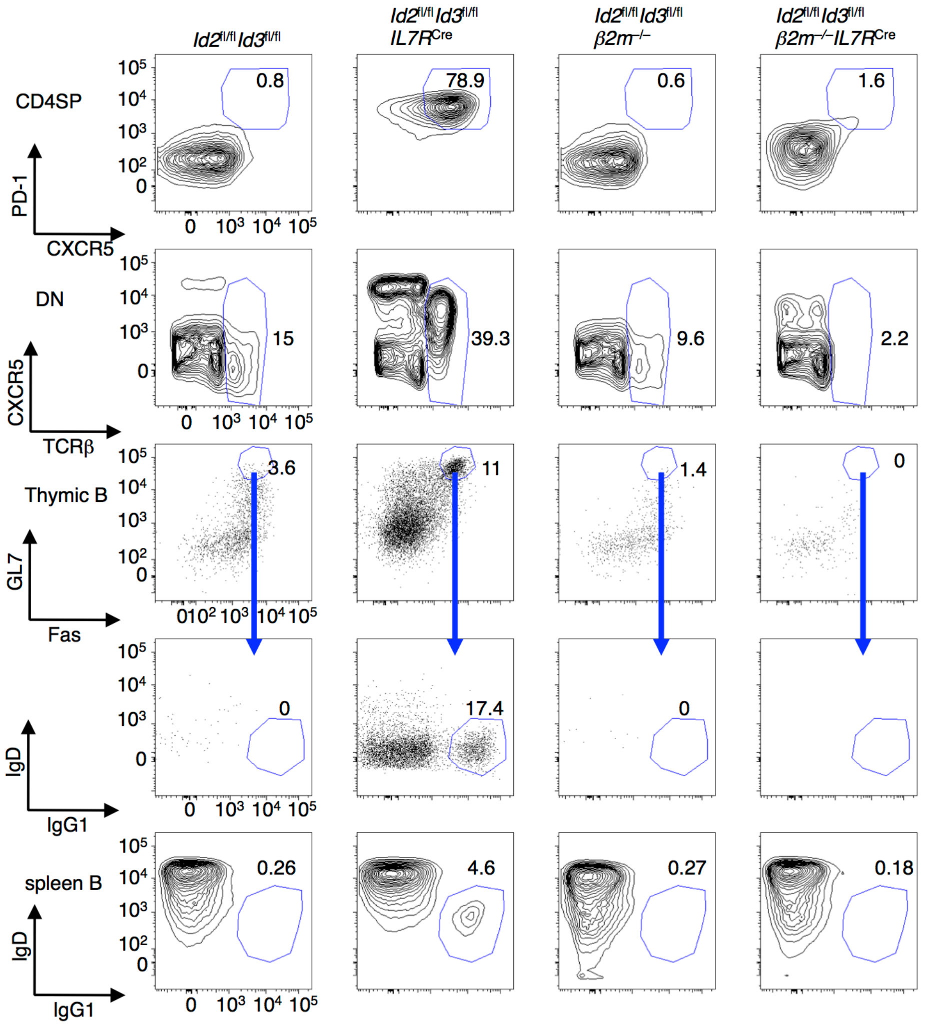

B

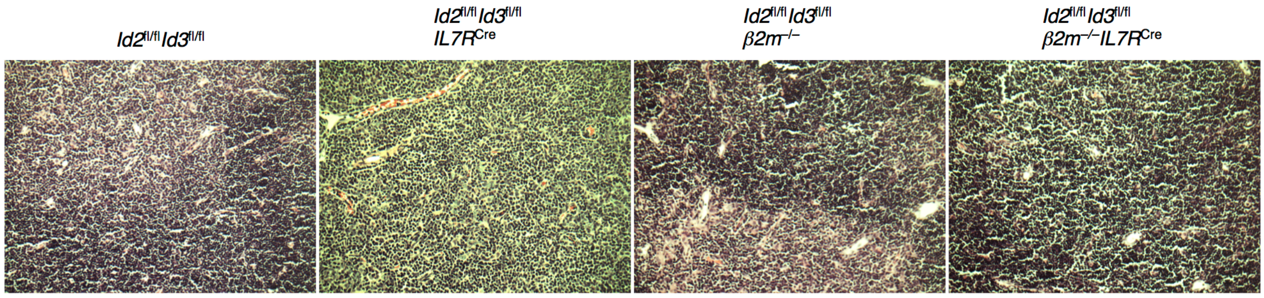

Figure S8. Related to Figure 4. β2m is required for the innate T_{FH} cells and IgG1-class switched GC B cells in thymus and IgG1 class switched B cells in spleen. (A) Flow cytometric analysis of CXCR5 versus PD-1 expression, gated on CD4SP cells, TCRβ versus CXCR5 expression gated on DN cells, Fas versus GL7 expression gated on thymic B cells, IgG1 versus IgD gated on GC B cells in thymus and splenic B cells. (B) Representative H&E staining of thymus. Original magnification: x200.

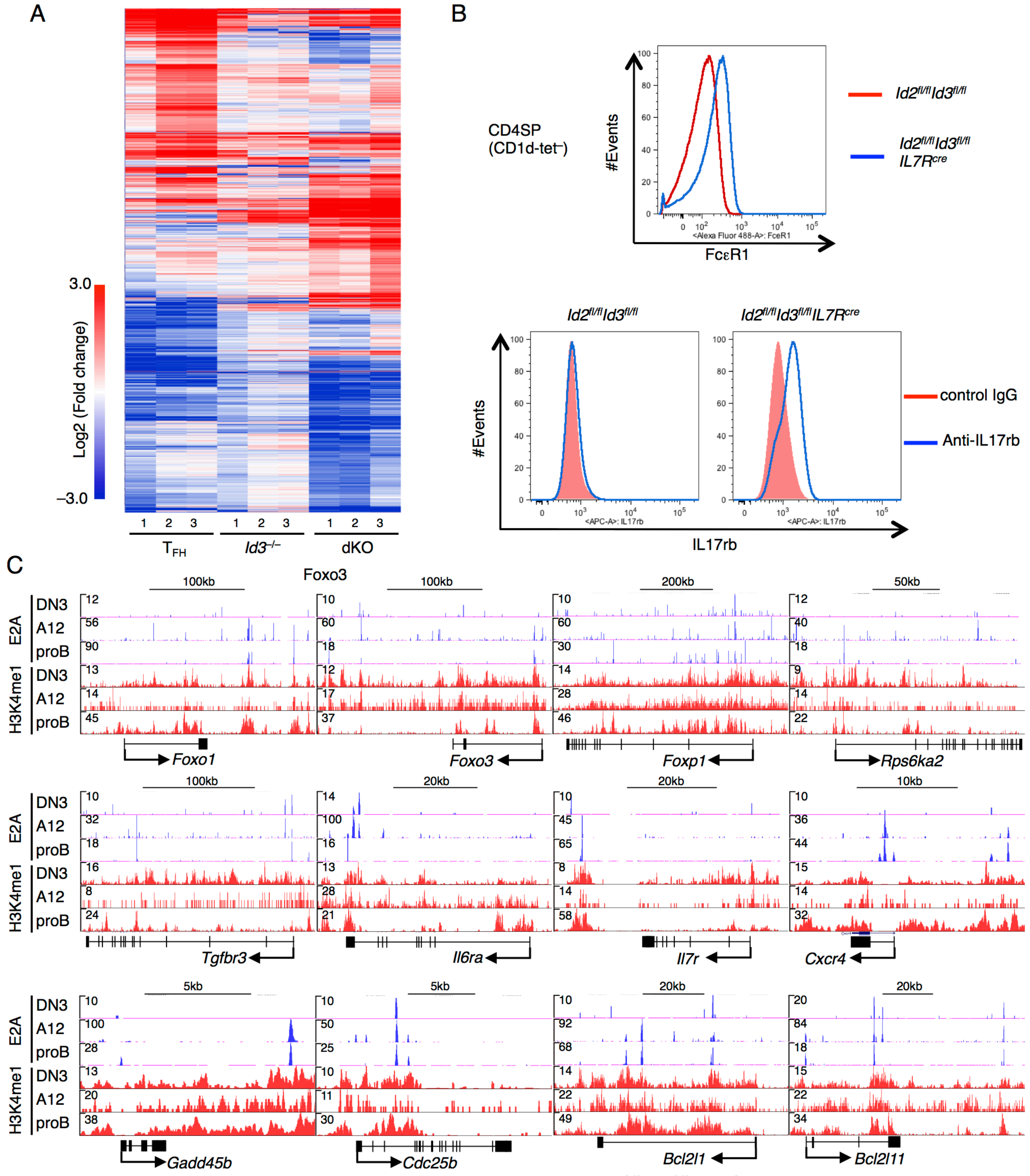

Figure S9. Related to Figure 5. Gene expression signatures of *Id2*$^{fl/fl}$*Id3*$^{fl/fl}$*dlck*^{Cre} CD4SP cells and E2A regulated loci. (A) Heatmap is displayed for significantly differentially expressed genes in T_{FH} cells, *Id3*$^{-/-}$ CD4SP and *Id2*$^{fl/fl}$*Id3*$^{fl/fl}$*IL7R*^{Cre} CD4SP cells, compared to control CD4SP cells (3601 genes; > twofold, *P* <0.05). (B) Flow cytometric analysis of FcεR1 and IL-17rb expression, gated on CD4SP ($CD4^{+}CD8^{-}$ $TCR\beta^{hi}$CD1d-tet^{-}) cells. (C) E2A occupancy and deposition of H3K4me1 across the *Foxo1, Foxo3, Foxp1, Rps6ka2, Tgfbr3, Il6ra, Il7r, Cxcr4, Gadd45b, Cdc25b, Bcl2l1, and Bcl2l11* loci in DN3, A12 and pro-B cells. Numbers in plots indicate total tags observed. DN3 cells; *Rag2*$^{-/-}$ thymocytes, A12 cells; *E2a*$^{-/-}$ E47 reconstituted T cell line, pro-B cells; *Rag2*$^{-/-}$ bone marrow B cells. Arrows indicate transcriptional start site and direction of transcription.

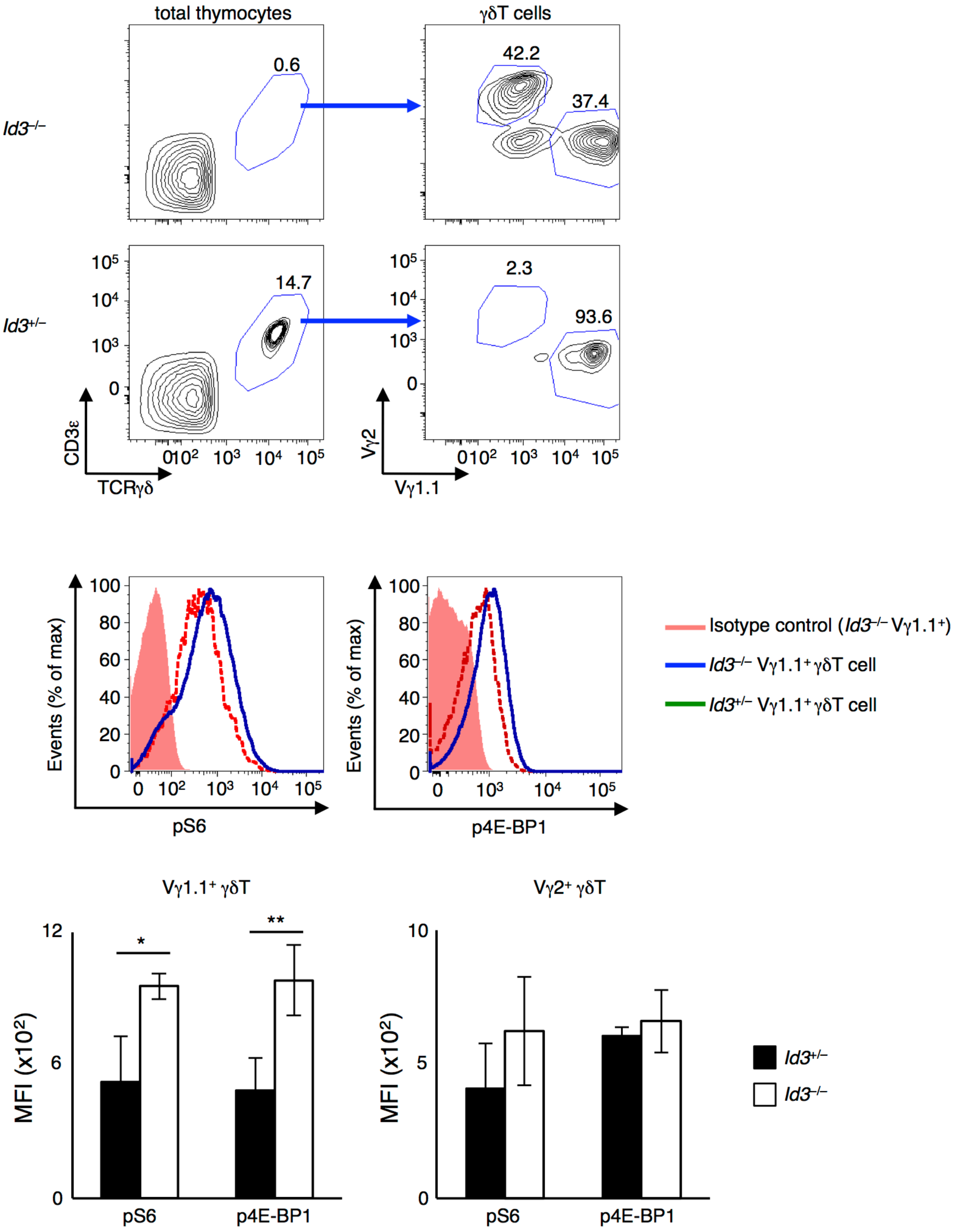

Figure S10. Related to Figure 6. Higher level of mTORC1 activity in Vγ1.1+ innate γδT cells, but not in Vγ2+ cells, in *Id3*−/− thymus.

Flow cytometric analysis of TCRγδ versus CD3ε expression in total thymocytes, and Vγ1.1 versus Vγ2 expression gated on γδT cells, derived from *Id3*+/− and *Id3*−/− thymus (top). Phospho-S6 and -4E-BP1 expression gated Vγ1.1+ γδT cells derived from *Id3*+/− and *Id3*−/− thymus (middle). Graph shows the level of phosphorylated S6 and 4E-BP1, presented as MFI. Data are representative of three independent experiments (mean ± s.d.; n = three biological replicates). ★, $P < 0.05$, ★★, $P < 0.01$ (Student's *t* test).

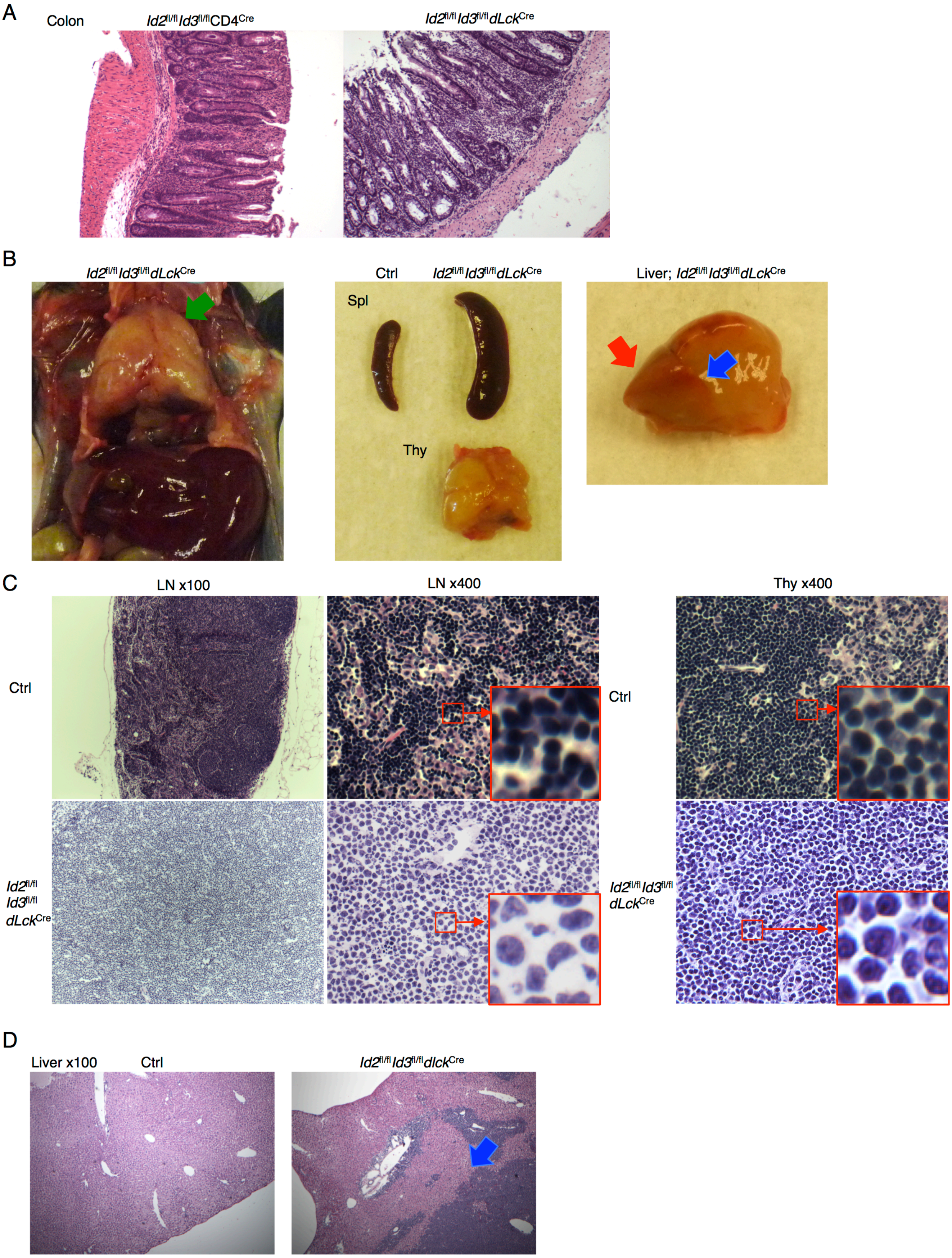
A
Colon
Id2$^{fl/fl}$Id3$^{fl/fl}$CD4^{Cre}
Id2$^{fl/fl}$Id3$^{fl/fl}$dLck^{Cre}
B
Id2$^{fl/fl}$Id3$^{fl/fl}$dLck^{Cre}
Ctrl
Id2$^{fl/fl}$Id3$^{fl/fl}$dLck^{Cre}
Spl
Thy
Liver; Id2$^{fl/fl}$Id3$^{fl/fl}$dLck^{Cre}
C
LN x100
LN x400
Thy x400
Ctrl
Ctrl
Id2$^{fl/fl}$ Id3$^{fl/fl}$ dLck^{Cre}
Id2$^{fl/fl}$Id3$^{fl/fl}$ dLck^{Cre}
D
Liver x100
Ctrl
Id2$^{fl/fl}$Id3$^{fl/fl}$dlck^{Cre}

E

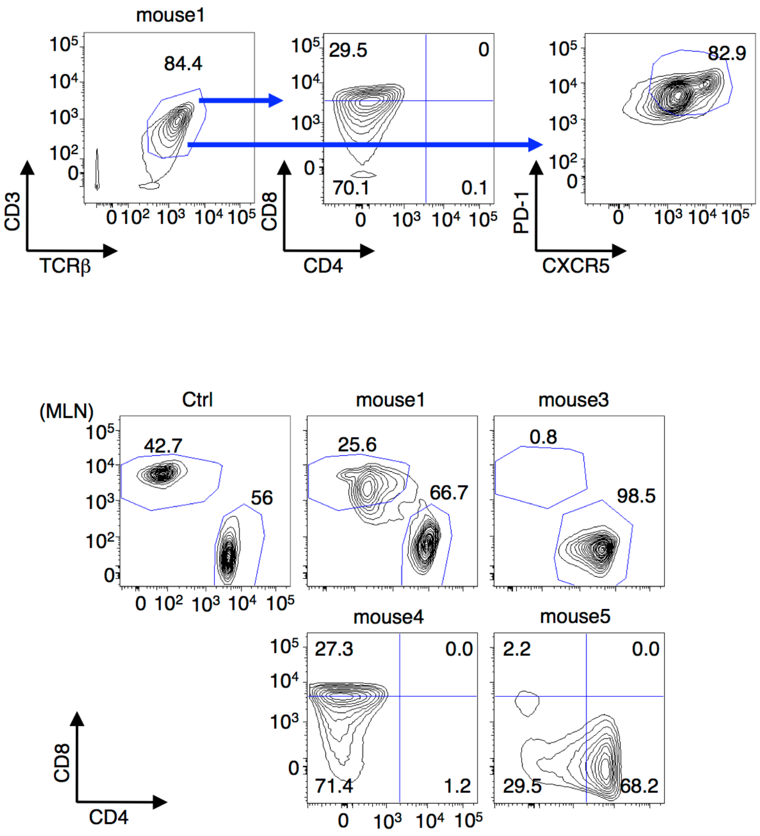

Figure S11. Related to Figure 7. Histological analysis of T cell lymphoma in *Id2*$^{fl/fl}$*Id3*$^{fl/fl}$*IL7R*^{Cre}/*CD4*^{Cre}/*dLck*^{Cre} mice.

(A) H&E staining of large intestine derived from *Id2*$^{fl/fl}$*Id3*$^{fl/fl}$*CD4*^{Cre}/*dLck*^{Cre} mice. Original magnification: x100. (B) Images of thymus, spleen, and liver derived from 12-month-old *Id2*$^{fl/fl}$*Id3*$^{fl/fl}$*dLck*^{Cre} mouse. Green arrow indicates thymic lymphoma. Blue arrow indicates infiltration of lymphoma cells in the liver. Red arrow indicates normal liver tissue. (C) H&E staining of LNs and thymus. (D) H&E staining of liver isolated from control and *Id2*$^{fl/fl}$*Id3*$^{fl/fl}$*dLck*^{Cre} mice, as seen in Figure S11B. Blue arrow indicates the border between lymphoma cell invasion and normal liver tissue. (E) Flow cytometric analysis of TCRβ versus CD3ε expression in thymic lymphoma cells, CD4 versus CD8, CXCR5 versus PD-1 expression gated on TCRβ^{+}CD3ε^{+} lymphoma cells (top). CD4 and CD8 expression gated on TCRβ^{+}CD3ε^{+} T cells (Ctrl) and lymphoma cells derived from *Id2*$^{fl/fl}$*Id3*$^{fl/fl}$*CD4*^{Cre}/*dLck*^{Cre} mesentric LNs.

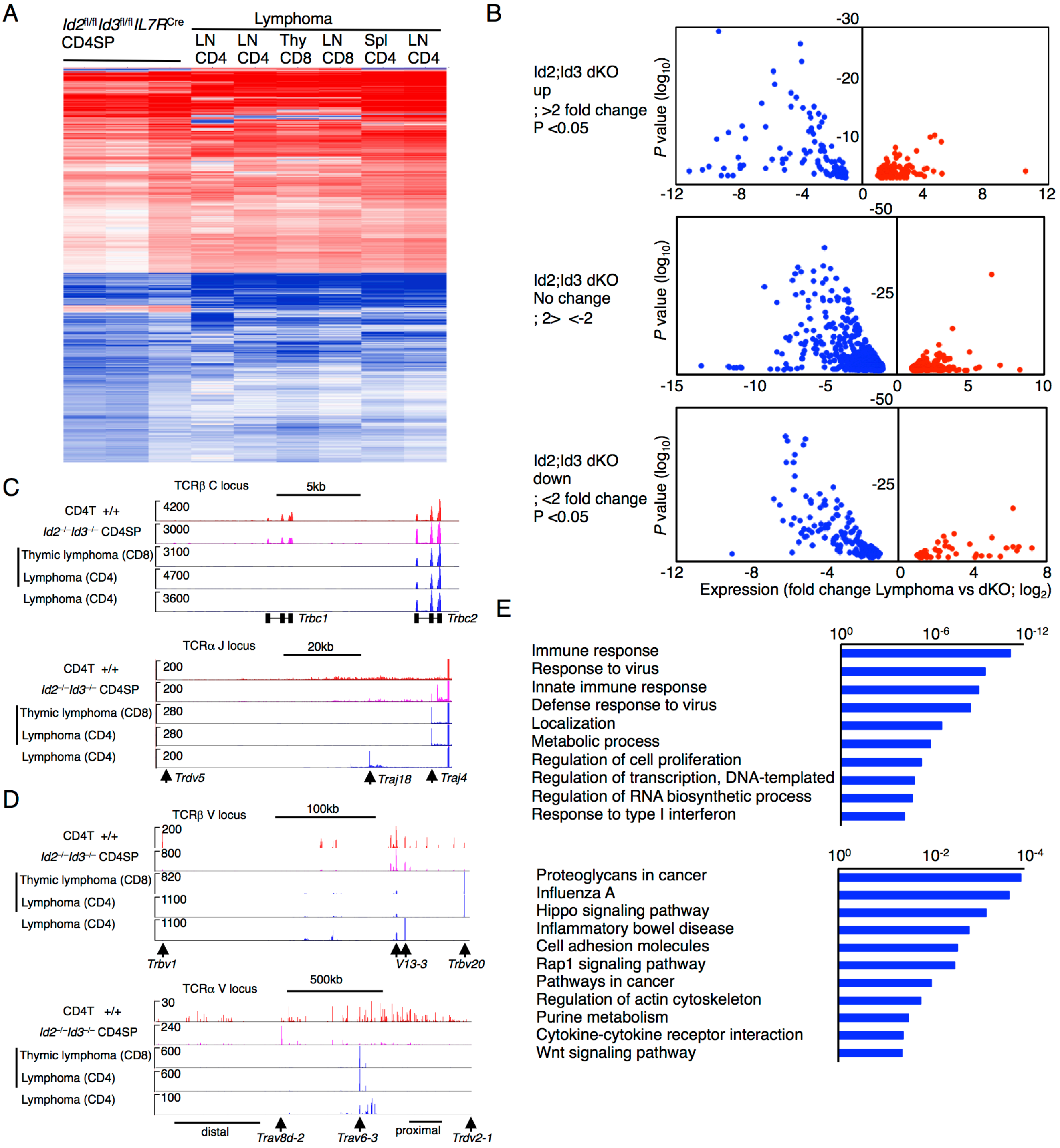

Figure S12. Related to Figure 7. Transcription signatures of *Id2*- and *Id3*-deficient T cell lymphomas. (A) Heatmap is displayed for significantly differentially expressed genes between $Id2^{fl/fl}Id3^{fl/fl}IL7R^{Cre}$ CD4SP cells and lymphoma cells (2122 genes; >twofold, $P<0.05$, any >10). (B) Volcano plots of RNA-seq analysis. Genes significantly differentially expressed between $Id2^{fl/fl}Id3^{fl/fl}IL7R^{Cre}$ CD4SP cells and lymphoma cells were further compared to control CD4SP cells, control CD4 T cells, and CD8 T cells. Then, those genes were classified into three groups, based on the expression in $Id2^{fl/fl}Id3^{fl/fl}IL7R^{Cre}$ CD4SP cells. Top; upregulated (>2-fold), middle; not changed (2> >0.5), bottom; (<0.5), in $Id2^{fl/fl}Id3^{fl/fl}IL7R^{Cre}$ CD4SP compared to control CD4SP cells as seen in Figure 5A. (C-D) RNA-seq analysis across *Trbc1/Trbc2* and *Traj* loci (C) and TCRβ V, and TCRα V loci. (E) Selected biological process GO (Gene Ontology) terms (top), KEGG pathways (bottom) and their associated *P*-values are shown.

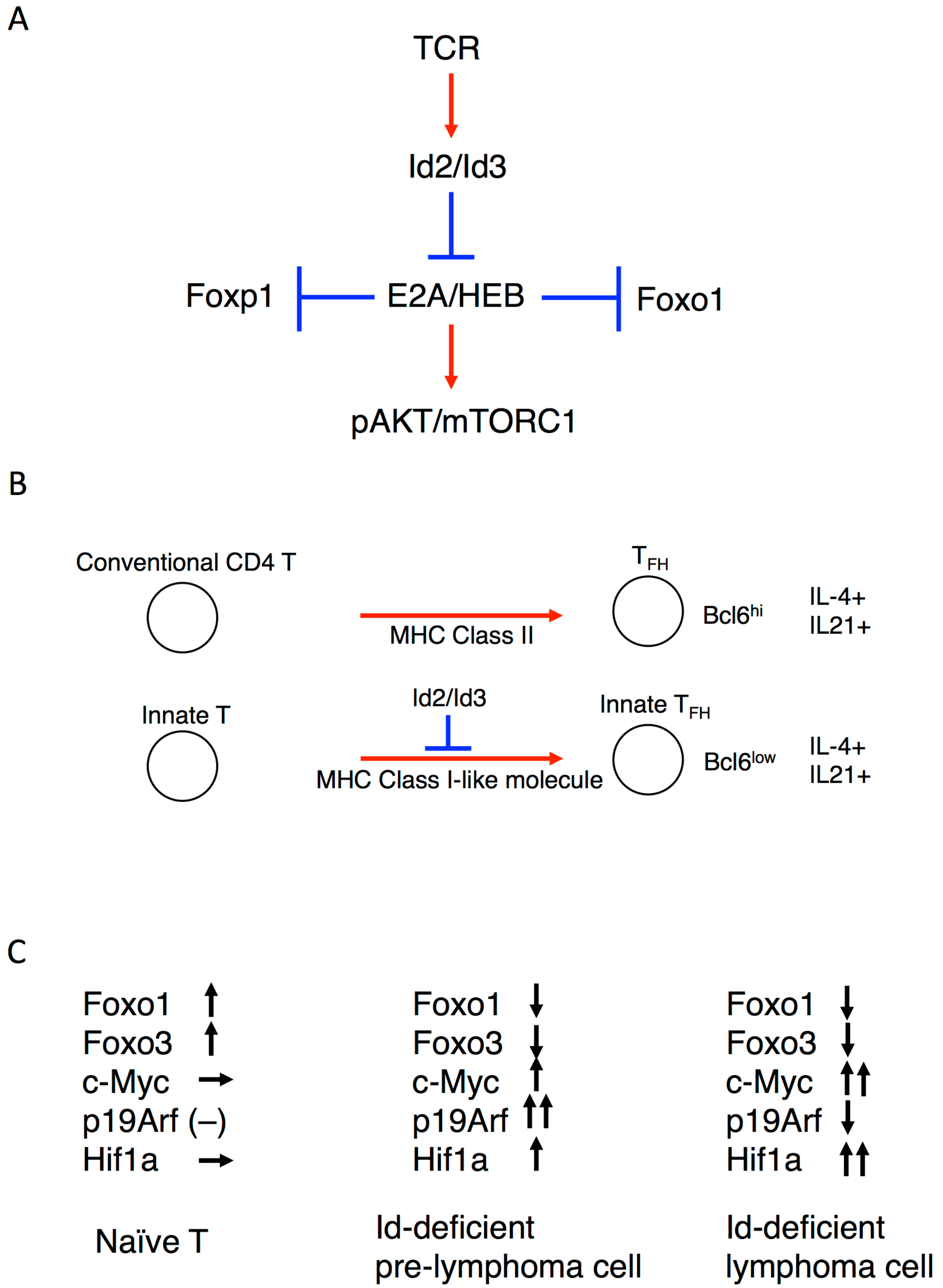

Figure S13. Related to Discussion. Models depicting the roles of Id2 and Id3. (A) Genetic circuitry involving Id-proteins and the AKT-FoxO-mTOR pathway. (B) Schematic diagram that displays the role of Id2 and Id3 in suppressing the development of innate variant T_{FH} cells. (C) Intermediate steps in Id-deficient lymphoma genesis characterized by modulating the expression of genes associated with lymphoid cell growth and malignancy.

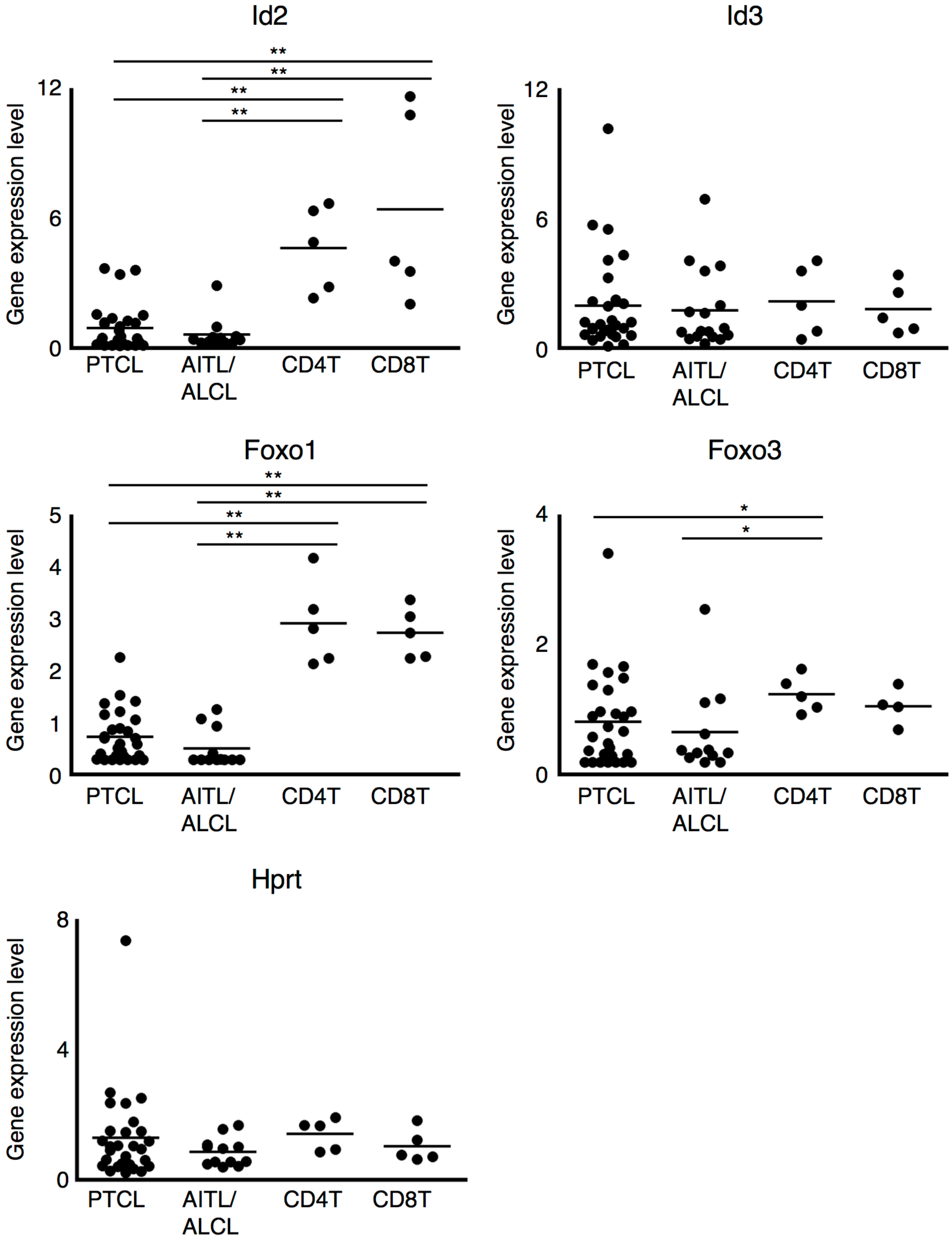

Figure S14. Related to Discussion. *Id2*, *Id3*, *Foxo1*, *Foxo3*, and *Hprt* expression in human T cell lymphoma. PTCL; peripheral T cell lymphoma / unspecified, AITL; Angioimmunoblastic T lymphoma, ALCL; Anaplastic large cell lymphoma. The expressions of selected genes were performed by using microarray data previously reported (Picculga et al. 2007)
